# Extra-Small Gold Nanospheres Decorated With a Thiol Functionalized Biodegradable and Biocompatible Linear Polyamidoamine as Nanovectors of Anticancer Molecules

**DOI:** 10.3389/fbioe.2020.00132

**Published:** 2020-03-04

**Authors:** Nora Bloise, Alessio Massironi, Cristina Della Pina, Jenny Alongi, Stella Siciliani, Amedea Manfredi, Marco Biggiogera, Michele Rossi, Paolo Ferruti, Elisabetta Ranucci, Livia Visai

**Affiliations:** ^1^Department of Molecular Medicine (DMM), Biochemistry Unit, Center for Health Technologies (CHT), UdR INSTM University of Pavia, Pavia, Italy; ^2^Department of Occupational Medicine, Toxicology and Environmental Risks, Istituti Clinici Scientifici Maugeri S.p.A, IRCCS, Pavia, Italy; ^3^Department of Chemistry and Industrial Chemistry, University of Pisa, UdR INSTM PISA, Pisa, Italy; ^4^Dipartimento di Chimica, Università degli Studi di Milano e CNR-ISTM, Milan, Italy; ^5^Dipartimento di Chimica, Università degli Studi di Milano, Milan, Italy; ^6^Department of Biology and Biotechnology, University of Pavia, Pavia, Italy

**Keywords:** cancer cell targeting, polyamidoamines, Trastuzumab, gold nanoparticles, breast cancer nanomedicine

## Abstract

Gold nanoparticles are elective candidate for cancer therapy. Current efforts are devoted to developing innovative methods for their synthesis. Besides, understanding their interaction with cells have become increasingly important for their clinical application. This work aims to describe a simple approach for the synthesis of extra-small gold nanoparticles for breast cancer therapy. In brief, a biocompatible and biodegradable polyamidoamine (named AGMA1-SH), bearing 20%, on a molar basis, thiol-functionalized repeat units, is employed to stabilize and coat extra-small gold nanospheres of different sizes (2.5, 3.5, and 5 nm in gold core), and to generate a nanoplatform for the link with Trastuzumab monoclonal antibody for HER2-positive breast cancer targeting. Dynamic light scattering, transmission electron microscopy, ultraviolet visible spectroscopy, X-ray powder diffraction, circular dichroism, protein quantification assays are used for the characterization. The targeting properties of the nanosystems are explored to achieve enhanced and selective uptake of AGMA1-SH-gold nanoparticles by *in vitro* studies against HER-2 overexpressing cells, SKBR-3 and compared to HER-2 low expressing cells, MCF-7, and normal fibroblast cell line, NIH-3T3. *In vitro* physicochemical characterization demonstrates that gold nanoparticles modified with AGMA1-SH are more stable in aqueous solution than the unmodified ones. Additionally, the greater gold nanoparticles size (5-nm) is associated with a higher stability and conjugation efficiency with Trastuzumab, which retains its folding and anticancer activity after the conjugation. In particular, the larger Trastuzumab functionalized nanoparticles displays the highest efficacy (via the pro-apoptotic protein increase, anti-apoptotic components decrease, survival-proliferation pathways downregulation) and internalization (via the activation of the classical clathrin-mediated endocytosis) in HER-2 overexpressing SKBR-3 cells, without eliciting significant effects on the other cell lines. The use of biocompatible AGMA1-SH for producing covalently stabilized gold nanoparticles to achieve selective targeting, cytotoxicity and uptake is completely novel, offering an important advancement for developing new anticancer conjugated-gold nanoparticles.

## Introduction

**FIGURE 1:**
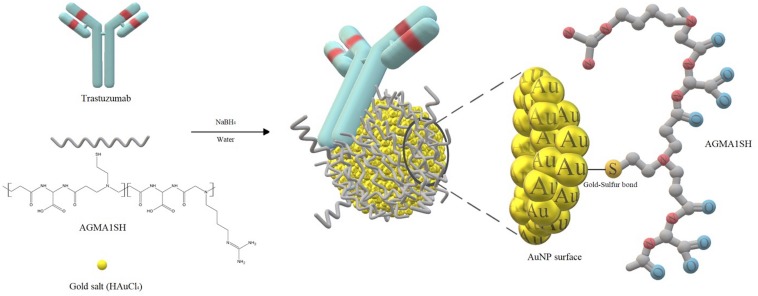
Schematic representation of AuNPs coated with AGMA1-SH and Trastuzumab.

Breast cancer is the most frequent and invasive cancer type in women (Rojas and Stuckey, 2016). There is an increasing demand for novel, efficient and specific diagnostic and medical tools to face the adverse side-effects of the traditional ones. Some invasive forms of breast cancer (i.e. locally advanced breast cancer) presented a twofold higher incidence than the others, suggesting an urgent improvement of Trastuzumab (Herceptin^TM^) delivery toward erbB2 tyrosine kinase receptor (HER-2) found overexpressed in ∼25%–30% of breast cancers (Sawaki et al., 2006). Engineered delivery systems based on gold nanoparticles (AuNPs) are particularly promising to address this problem. AuNP formulations are employed for a wide range of medical applications, including contrast and photothermal agents for computed tomography (CT) and tumor photothermal ablation, respectively (Mieszawska et al., 2013). Their chemical and physical properties, that span the broader visible to near-infrared, ensure them features such as low toxicity, high stability, easy synthesis and conjugation with cancer-specific biomolecules (Lee et al., 2014; Carnovale et al., 2016). It is generally agreed that physicochemical differences in nanoparticles such as particle size, shape, surface charge and surface coating could vary the way particles are recognized, processed and excreted by the body (Carnovale et al., 2016). Notably, the molecular rules governing these properties are poorly understood, and the different set-up conditions used in the different laboratories (such as cell types, dosage schedule, measurement methods) make it difficult to drawing valid and conclusive information. Experimental and theoretical studies stated that the optimal size for AuNPs cellular uptake ranges from 25 to 50 nm, since it stimulates efficiently membrane wrapping and receptor-ligand interaction to drive the NPs into the cell (Gao et al., 2005; Jiang et al., 2008; Dreaden et al., 2012; Panariti et al., 2012; Saw et al., 2018). Surface charge can also have an effect on the cellular uptake (Zhang et al., 2015). Interacting with negative cell membranes, the electropositive nanoparticles exhibited a higher cellular uptake efficiency compared to electronegative ones. Conjugation of AuNPs with targeting molecules effectively improves the tumor target delivery (Paciotti et al., 2004). Several studies reported that Trastuzumab could be successfully immobilized on gold nanoparticles to improve their interactions with SKBR3 breast cancer cells, overcome Trastuzumab resistance and detect breast cancer (Hainfeld et al., 2006; Bickford et al., 2008; Chattopadhyay et al., 2010; Carpin et al., 2011; Lee et al., 2014).*In vitro* experiments indicated that, while human skin cells proliferated in the presence of Trastuzumab-conjugated gold nanoparticles, most of the breast cancer cells died (Rathinaraj et al., 2015). Despite the broad interest surrounding gold-based nanosystems, reproducibility, toxicity and excretion concerns limit their clinical translations (Choi et al., 2007; Lewinski et al., 2008; Tam et al., 2010). Indeed, currently no gold nanoparticles have yet been approved by the FDA agency. Different biodegradable polymers were tested for assembling and coating gold nanoparticles clusters (Tam et al., 2010), while minimizing immunogenicity reactions. Cheheltani et al. (2016) proposed a small, excretable AuNP-based platform, encapsulated into biodegradable poly di(carboxylatophenoxy)phosphazene (PCPP) nanospheres. A study by Tam et al. (2010) reported polymer/inorganic nanoclusters combining the imaging contrast and therapeutic capabilities with the biodegradability of a polymer stabilizer. Linear polyamidoamines (PAAs) have recently emerged as promising tools for drug delivery as they offer key advantages due to their ease of formulation and biodegradability (Ferruti et al., 2005; Jacchetti et al., 2008; Ferruti, 2013; Mauro et al., 2013). PAAs were previously investigated as anticancer drug carriers (Lavignac et al., 2009). In particular, the PAA nicknamed AGMA1 can be used as a potential non-viral, non-toxic and efficient vector for the intracellular delivery of siRNA and DNA (Cavalli et al., 2010; Cavalli et al., 2017). Interestingly, AGMA1, containing tert-amine, carboxyl and guanidine groups, whose repeat unit is reminiscent of the arg-gly-asp (RGD) peptide motif (Franchini et al., 2006), a well-known fibronectin sequence mediating cell attachment, can act as an excellent cell adhesion and proliferation substrate (Gualandi et al., 2016). For *in vivo* applications, gold-based nanosystems should be larger than 6 nm in diameter to ensure long blood circulation, hence accumulation in diseased tissues but slowly breaking down into sub-6 nm components for rapidly excretion via the kidneys (Arruebo et al., 2007; Choi et al., 2007). The goal of the present study was to develop more efficient gold nanoparticles for therapeutic use. To this purpose, a biocompatible and biodegradable polyamidoamine bearing 20%, on a molar basis, randomly distributed SH pendants (AGMA1-SH, indicated also as “P”) was employed to stabilize AuNPs of different sizes, that is 2.5, 3.5, and 5 nm in Au core (Au@P), decorated with Trastuzumab (Au@PT), whose hydrodynamic diameter was suitable for a cellular uptake (Figure 1). AGMA1, besides being a biocompatible and biodegradable polymer, was found to be easily internalized in cells and, therefore, it can be expected to facilitate the AuNPs cellular uptake (Franchini et al., 2006; Cavalli et al., 2010), whereas thiol groups are supposed to stabilize the AGMA1-gold bond by the renowned S-Au soft-soft interaction. Once tested *in vivo*, it is reasonable to speculate that, because of the AGMA1 biodegradability, the small Au core component (ranging from 2.5 to 5 nm) may be the only one to be extruded by the urinary system. In this work, the impact size of Au@P nanoparticles on the interactions with both breast cancer cells, and healthy cells was systemically studied. The cytotoxicity and cellular uptake of all nanoparticles were studied by MTT and ICP-MS tests. Western blot analysis and cellular uptake studies by means of specific endocytosis inhibitors were further carried out to explore the interaction with and mechanism of internalization of Au@PT by SKBR-3 target cells.

## Materials and Methods

### Reagents

4-Aminobutylguanidine sulfate (97%), lithium hydroxide monohydrate (99%), cystamine hydrochloride (96%), 2-mercaptoethanol (99%) and calcium chloride (97%) were purchased from Sigma-Aldrich and used as provided. 2,2-Bis(acrylamido)acetic acid (96%) was synthesized as previously described (Ferruti et al., 1999). HAuCl_4_ solution (composition: 17 wt.% Au; concentration: 30 wt.% in dilute HCl) was purchased from Sigma-Aldrich and diluted with water to achieve a final Au concentration of 10 mg/mL. NaBH_4_ (>96% pure), purchased from Sigma-Aldrich, was employed as the reducing agent for Au^3+^ to colloidal gold dispersion (10 mg/mL). Activated carbon Vulcan XC72R (specific area = 254 m^2^ g^–1^, pore volume = 0.19 mL g^–1^) from CABOT was used as a supporting material for colloidal samples for XRPD analysis. MilliQ water obtained by an Academic A-10 Millipore apparatus was employed as a solvent for all aqueous solutions. Trastuzumab (Herceptin^®^, Roche) was kindly provided by the Chemotherapic section of Policlinico San Matteo (Pavia). For Western blot analyses, primary mouse monoclonal anti β-actin was purchased from Santa Cruz Biotechnology, mouse monoclonal anti-ERK1/2 and rabbit polyclonal anti-phospho ERK1/2 (Thr 202/204) from Abcam, rabbit monoclonal anti-AKT and rabbit polyclonal anti-phospho -AKT (Ser 473) from Cell Signaling Technologies (Danvers, MA, United States) and rabbit monoclonal anti-BAX and anti-BCL-XL from Thermo Fisher Scientific. Horseradish peroxidase (HRP)-conjugated secondary antibodies anti-mouse and anti-rabbit (Dako) anti-human (Tebu-bio) were used, and detection was performed by enhanced chemiluminescent substrate (ECL) solutions (Pierce Thermo Fisher Scientific, Rockford, IL, United States).

### Instruments

^1^H and ^13^C NMR spectra were run on a Brüker Advance 400 spectrometer operating at 400.132 (^1^H) and 100.623 (^13^C) MHz. Size exclusion chromatography (SEC) traces were obtained with a Knauer Pump 1000 equipped with a Knauer Autosampler 3800, TSKgel G4000 PW and G3000 PW TosoHaas columns connected in series, Light Scattering (LS) Viscotek 270 Dual Detector, UV detector Waters model 486, operating at 230 nm, and a refractive index detector Waters model 2410. The mobile phase was a 0.1 M Tris buffer pH 8.00 (0.05 M with 0.2 M sodium chloride (Bignotti et al., 1994). The operational conditions were: sample concentration 20 mg/mL; flow rate 1 μL/min; injection volume 20 μL; column dimensions 300 × 7.5 mm^2^, temperature 25°C. The instrument constants were determined using PEO 19 kDa as a narrow polymer standard. All samples were filtered through a 0.2 μm syringe Whatman filter before measurements.

### Synthesis of AGMA1-SH

2,2-Bis(acrylamido)acetic acid (4.128 g, 20.0 mmol) and lithium hydroxide monohydrate (1.53 g, 36.0 mmol) were dissolved in water (18 mL) and 4-aminobutylguanidine (agmatine) sulfate (3.765 g, 16.0 mmol) was added under vigorous stirring. The reaction mixture was heated up to 40°C until dissolution and kept under nitrogen atmosphere at room temperature with gentle stirring for 1 day (d). After this time, the reaction mixture was cooled down to room temperature and cystamine dihydrochloride (0.469 g, 2.0 mmol) and lithium hydroxide monohydrate (0.170 g, 4 mmol) were added. The resultant mixture was kept at room temperature for 10 days until a soft transparent hydrogel was obtained. Then water (40 mL) and excess mercaptoethanol (8.0 mL) were added until a clear solution was obtained. This was diluted with water (100 mL), acidified to pH 4.5 with 37% hydrochloric acid and then ultrafiltered through a membrane with nominal molecular weight cut-off 3000. The final product was retrieved by freeze-drying the retained fraction. Yield 68%, $\bar{\text{M}}\,n=8400$, PDI = 1.25.

^1^H NMR (D_2_O): δ (ppm) = 1.52–1.60 (br, NCH_2_CH_2_CH_2_); 1.71–1.77 (br, NCH_2_CH_2_); 2.64–2.76 (br, NHCOCH_2_); 2.92–2.94 (m, CH_2_S); 3.08–3.20 (m, NCOCH_2_CH_2_N); 3.34–3.47 (br, NCH_2_CH_2_CH_2_ and NCH_2_CH_2_S); 5.51–5.56 (s, CHCOOH).

^13^C NMR (D_2_O): d (ppm) = 18.5 (CH_2_S); 22.3 (NCH_2_ CH_2_CH_2_); 25.0 (NCH_2_CH_2_); 29.0 (NHCOCH_2_); 40.4 (CH_2_ CH_2_NH); 49.1 (NCH_2_CH_2_CONH); 52.5 (NCH_2_CH_2_CH_2_CH_2_ NH); 54.8 (NCH_2_CH_2_S); 56.0 (CHCOOH); 155.1 (H_2_NC = NH); 171.3 (NHCO); 173.5 (COOH).

### Synthesis of Gold Nanoparticles Decorated With AGMA1-SH and Trastuzumab

Three differently sized colloidal samples of AGMA1-SH-coated gold nanoparticles decorated with Trastuzumab (Herceptin^®^), were prepared by varying gold solution concentration from 20 ppm to 400 ppm. The samples were labeled as 2.5Au@PT, 3.5Au@PT, and 5Au@PT, where the number indicates the average diameter of gold *core* (2.5, 3.5, and 5.0 nm, respectively), P means the polymer (AGMA1-SH) and T the antibody drug (Trastuzumab). Accordingly, the following parameters were optimized to produce stable colloidal nanosystems over time: gold solution concentration (20 ppm for 2.5Au@PT, 200 ppm for 3.5Au@PT, and 400 ppm for 5Au@PT), AGMA1-SH:Au:Trastuzumab ratio (1wt.:1wt.:1wt.) and NaBH_4_:Au ratio (1wt.:1wt.). More in detail, proper aliquots of AGMA1-SH aqueous solution (10 mg/mL) were added under stirring to defined volumes of HAuCl_4_ aqueous solution (10 mg/mL Au) and MilliQ^®^ water at room temperature. Established amounts of freshly prepared NaBH_4_ aqueous solution (10 mg/mL) were rapidly added under vigorous stirring to reduce Au^3+^ to Au^0^ and, hence, produce light-tea to reddish colored colloidal dispersions. Finally, proper volumes of Trastuzumab aqueous solution (20 mg/mL) were poured drop wise. Only in the case of the 5Au@PT sample, sodium borohydride was added prior to the polymer in order to allow gold nanoparticles to grow up to 5.0 nm. Supplementary Table S1 reports the established aliquots for each component. At the end of reaction, to remove the excess of synthesis reagents or/and to eliminate unbound antibody, each AuNPs solution was purified by dialysis for 24 h in a Float-A-Lyzer Spectra/Por G2 (MWCO of 100 kDa for Au@PT) under stirring at 4°C.

### Synthesis of Gold Nanoparticles Decorated With AGMA1-SH or Trastuzumab

Three differently sized AuNP@AGMA1-SH colloidal samples without the drug (Trastuzumab) and three differently sized AuNP@Trastuzumab colloidal samples without the polymer (AGMA1-SH) were synthesized adopting the aforementioned procedure. The following parameters were optimized in order to produce stable colloidal nanosystems: gold solution concentration (20 – 400 ppm), AGMA1-SH:Au or Trastuzumab:Au ratios (1wt.:1wt.) and NaBH_4_:Au ratio (1wt.:1wt.). The samples were labeled as 2.5Au@P, 3.5Au@P, 5Au@P and 2.5Au@T, 3.5Au@T, 5Au@T respectively. Supplementary Table S1 reports the established aliquots for each component. At the end of reaction, to remove the excess of synthesis reagents each AuNPs solution was purified by dialysis for 24 hours (h) in a Float-A-Lyzer Spectra/Por G2 (MWCO 10 kDa for Au@P and 100 kDa for Au@T, respectively) under stirring at 4°C. As evinced by microscopic analyses (TEM), AuNPs were synthesized in uniform spherical sizes. Hence, by using the simple geometrical model of spherical particles (Eq. 1–6), the theoretical Trastuzumab molecule to gold nanoparticle ratio (T/AuNP) could be determined for the three differently sized nanosystems. Accordingly, T/AuNP = 0.65 for ‘2.5Au’ samples, 1.79 for ‘3.5Au’ samples and 5.21 for ‘5Au’ samples. In addition, extrapolating the fraction of gold atoms lying at the surface (*gold dispersion* from Supplementary Figure S1) led to the theoretical Trastuzumab molecule to gold surface atom ratio (T/surface Au) for the three differently sized samples:

T/surface Au = 0.0025 for ‘2.5Au’ samples, 0.0039 for ‘3.5Au’ samples and 0.0054 for ‘5Au’ samples.

(1) $$volumeAuNP=\frac{4}{3}\pi r^{3}\;(r\text{is Au nanoparticle radius}=1.25,1.75,\text{and 2.5 nm respectively})$$

(2) $$mass\;AuNP\;=\;volume\;AuNP\;\times \rho \left(\rho \text{is}\;\text{Au}\;\text{density}=19\text{.3}\;{\text{g/cm}}^{3}\right)$$

(3) $$n\,u\,m\,b\,e\,r\,A\,u\,N\,P=\frac{m\,a\,s\,s\,A\,u\,i\,n\,t\,h\,e\,s\,a\,m\,p\,l\,e}{m\,a\,s\,s\,A\,u\,N\,P}$$

(4) m⁢o⁢l⁢e⁢s⁢A⁢u⁢N⁢P=m⁢a⁢s⁢s⁢A⁢u⁢N⁢P/a⁢t⁢o⁢m⁢i⁢c⁢w⁢e⁢i⁢g⁢h⁢t⁢A⁢u

(5) $$a\,t\,o\,m\,s\,A\,u\,N\,P=m\,o\,l\,e\,s\,A\,u\,N\,P\times A\,v\,o\,g\,a\,d\,r\,o^{\prime }\,s\,n\,u\,m\,b\,e\,r$$

surfaceatomsAuNP=golddispersion(%)×atomsAuNP

(6) (Golddispersionis 55% for ‘2.5Au’, 35%for ‘3.5Au’ and 25% for ‘5Au’)

### Physicochemical Characterization

Dynamic light scattering (DLS) and Z-potential analyses were carried out on 1 mg/mL nanoparticle aqueous suspensions prepared using MilliQ water, with a Malvern Zetasizer NanoZS instrument equipped with a laser fitted at 532 nm and fixed 173° scattering angle. Before analyses, samples were filtered through a 0.2 μm syringe Whatman filter. The solution pH was adjusted to the selected value, using 0.1 M HCl or 0.1 M NaOH aqueous solutions. Measurements were performed in triplicate, and the reported values averaged over of 10 runs. Regarding DLS measurements on AGMA1-SH-coated gold nanoparticles decorated with Trastuzumab (Au@PT), AGMA1-SH-coated gold nanoparticles (Au@P) and Trastuzumab-coated gold nanoparticles (Au@Trastuzumab), 20 mg/L aqueous suspensions were analyzed. X-ray powder diffraction (XRPD) analyses were performed employing a Rigaku D III-MAX horizontal-scan powder diffractometer with Cu Kα radiation (λ = 1.5418 Å) to determine the average size of gold crystallites using the *Scherrer* equation (Patterson, 1939). The *Bragg* angle was rotated in the 34°– 43° *2*θ interval and the mean diameter *d* of gold *core* was determined considering the peak at *2*θ = 38.5°, typical of metallic gold. All the colloidal samples were immobilized on XC72R carbon to obtain the theoretical amount of 1% Au. UV-vis spectra were collected in the 200–800 nm region using a HP 8453 diode array instrument on colloidal samples (20 ppm Au) for determining the plasmon resonance band (PRB) of gold nanoparticles detected near 500–530 nm in the 2–10-nm-diameter range.

### Transmission Electron Microscopy

TEM analysis was performed by using a JEOL JEM 3010 operating at a 300-kV acceleration voltage. Briefly, 10 μL of each type of gold nanoparticle suspension in water (20 μg/mL) deposed on Parlodion^TM^ membranes.

### Efficiency of Trastuzumab Conjugation

The efficiency of Trastuzumab conjugation to nanoparticles was assessed by indirect (Bicinchoninic Acid, BCA), and direct (dot-blot) approaches as previously described (Liu et al., 2010; Zhou et al., 2015; Di Francesco et al., 2018; Codullo et al., 2019). In short, both Au@PT and Au@T nanoparticles suspensions (20 μg/mL) were centrifuged at 3000 × *g* for 15 min, then the supernatants were collected for determine the unbound Trastuzumab concentration by BCA assay (Pierce Biotechnology Inc., Rockford, IL, United States), while the pellets were re-suspended in 1 mL of MilliQ water for dot blot assay.

#### BCA Assay

The unbound content in the resulting solution was determined by BCA assay according to the specifications of the manufacturer. The unbound amounts were calculated from properly drawn Trastuzumab calibration curve. Trastuzumab amount conjugated to nanoparticles was thus obtained via reduction of the amount in the supernatant from the initial amount. The percentage of conjugation efficiency (CE) was calculated as follows (Martínez-Jothar et al., 2019):

$$\%CE=\frac{\left(t\,o\,t\,a\,l\,a\,m\,o\,u\,n\,t\,o\,f\,T\,a\,d\,d\,e\,d\,\text{-}\,u\,n\,b\,o\,u\,n\,d\,T\right)}{t\,o\,t\,a\,l\,a\,m\,o\,u\,n\,t\,o\,f\,T\,a\,d\,d\,e\,d}*100$$

#### Dot Blot Assay

Different amounts of Trastuzumab-functionalized Au@P differently sized were spotted on nitrocellulose membrane Amersham Hybond ECL, GE Healthcare Life Sciences, Pittsburgh, PA, United States) and air-dried. 2.5Au@P, 3.5Au@P, 5Au@P as negative controls. In parallel, Au@T samples dot blot was also performed. The non-specific sites were blocked by soaking the membrane in 5% BSA in TBS containing 0.05% Tween 20 for 1 h at room temperature (RT). The membrane was then incubated using goat anti-human-HRP-conjugated antibody (1:1000) for 1 h at RT and after extensive washing developed with enhanced chemiluminescence reagents (LI-COR) and ImageQuant LAS4000 Imaging System (Ge Healthcare). The spots were then analyzed with ImageQuant TL software (Ge Healthcare) and the results were normalized to calibration curve containing known amounts of Trastuzumab.

### Circular Dichroism

The secondary structure of the Trastuzumab free (20 μg/mL) and Trastuzumab-conjugated gold nanoparticles (20 μg/mL) were evaluated with circular dichroism (CD). CD spectra were measured using Jasco J710 spectropolarimeter (Jasco Corp., Tokyo, Japan) at 25°C and in a 1 cm path-length quartz cell under the following conditions: 300–190 nm spectral range, 2 nm of bandwidth, 200 nm min-1 of scan speed and 3 accumulation. Data was processed using 10-point smoothing in Origin 6.0 (OriginLab Corporation, Northampton, MA, United States).

### *In vitro* Trastuzumab Release From Gold Nanoparticles Decorated With AGMA1-SH

2.5Au@PT, 3.5Au@PT, and 5Au@PT (20 μg) were suspended in 1 mL of MilliQ water, in a low-protein binding centrifuge tube, and kept at room temperature. At select time points (1 – 15 days), each type of nanoparticles suspension was centrifuged, the supernatant was collected and replaced with the same volume of fresh release medium (Colzani et al., 2018). The concentration of antibody in the supernatants was determined using the BCA Kit as described above. Standard curve of Trastuzumab was used to determine antibody concentrations in each sample. The results are expressed as the percentage of cumulative fraction of Trastuzumab released at each time point compared to the conjugated amount. The *in vitro* release of 2.5Au@T, 3.5Au@T, and 5Au@T was not analyzed due to the higher aggregation recorded immediately within the first days after their synthesis.

### Cell Types and Culture Conditions

HER2 positive breast adenocarcinoma cells (SKBR-3), ERα positive breast adenocarcinoma cells (MCF-7) used as cancer negative control, murine fibroblasts cells (NIH-3T3) used as negative control. All cell lines were obtained from the American Type Culture Collection (HTB85, ATCC, Manassas, VA, United States). After thawing, SKBR-3 cell line was cultured in McCoy’s 5A Medium Modified (Sigma-Aldrich), 10% of Fetal Bovine Serum (FBS, EuroClone), 1% of L-Glutamine (Lonza), 2% sodium pyruvate (Lonza), 0.4% antibiotics (Lonza) and 0.1% fungizone. MCF-7 cell line was cultured in Minimum Essential Medium Eagle (Sigma-Aldrich), 10% of Fetal Bovine Serum (EuroClone), 1% sodium pyruvate (Lonza), 1% Non-Essential Aminoacids (EuroClone), 1% Bovine Insulin (Sigma-Aldrich), 1% L-Glutamine (Lonza) and 0.4% antibiotics (Lonza). NIH-3T3 cell line was cultured in Dulbecco’s Modified Eagle Medium High Glucose 4.5 mg/mL (Sigma-Aldrich), 10% of Bovine Calf Serum (BCS, EuroClone) and 1% of L-Glutamine (Lonza). All cell lines were incubated at 37°C in 5% CO_2_, routinely trypsinized (Trypsin EDTA solution 1X, Lonza) after confluence, counted and seeded into wells.

### Stability of AuNPs Upon Incubation With Cell Culture Media by Dynamic Light Scattering

Prior to all biological investigations, each suspension of AGMA1-SH-coated gold nanoparticles decorated with Trastuzumab (2 μg/mL) was centrifuged and resuspended in the cell culture media appropriate for the corresponding cell line, containing 10% of serum, and incubated at physiological temperature (37°C), without shaking, to mimic cell culture conditions (Halamoda-Kenzaoui et al., 2015; Marişca and Leopold, 2019). Size change was tracked by serial DLS size measurements at 0 (directly after dispersion in the complex media) and after 4 h of incubation.

### Cell Viability

Cells were seeded at 1 × 10^4^ viable cells/well on 96-well plates and incubated for 24 h (to allow cells to attach to the well). After the culture medium removal, cells were incubated with media containing increasing concentration of each types of gold nanoparticles. At fixed incubation times, the quantitative 3-[4,5-dimethylthiazol-2-yl]-2,5 diphenyl tetrazolium bromide (MTT, Sigma Aldrich, St. Louis, MO, United States) assay was performed to assess the dehydrogenase activity, as an indicator of the metabolic state of cells, as previously described (Merli et al., 2018). The cell viability was expressed as percentage related to the control (untreated) set equal to 100%. At day 3 of incubation, a qualitative viability assay [fluorescein diacetate (FDA) assay] was performed on SKBR-3 untreated (ctrl), treated with T free (2 μg/mL), 2.5Au@PT (2 μg/mL) and 5Au@PT (2 μg/mL), as previously described (Bloise et al., 2013).

### Scanning Electron Microscopy (SEM)

For morphological evaluation all types of cells were seeded on plastic cell culture coverslip disks (Thermanox Plastic, Nalge Nunc International, Rochester, NY, United States), then incubated or not with nanoparticles suspensions (2 μg/mL) and treated as previously described (Bloise et al., 2013). Before observation, samples were sputter coated with gold and observed using a Field Emission Scanning Electron Microscope (FESEM Supra 25, Zeiss).

### Uptake Studies by Inductively Coupled Plasma Mass Spectrometry (ICP-MS)

All types of cell lines were plated (500000/well) on a 6-well plate and treated for 1 and 3 days with 2 μg/mL of each types of nanoparticles (2 mL volume added in each well). After treatments, cells were washed three times with PBS to eliminate the NPs which were not internalized in cells, trypsinized, counted and centrifuged in order to obtain the cellular pellet. The cellular pellet was treated with 1X RIPA buffer (diluting the 10X RIPA in sterile distilled water, EDM Millipore Corporation, Chemicon) in ice for 30 min, transferred in sterile Eppendorf and centrifuged at 13000 rpm for 15 min at 4°C. The pellet was separated from the supernatant and treated with 750 μL of freshly prepared aqua regia. The samples were diluted to 3 mL with bidistilled water and analyzed for the Au content with ICP-MS (ELAN DRC, Perkin Elmer). The nanoparticles uptake efficiency per cell was calculated as previously described (Gunduz et al., 2017): *Uptake efficiency (%)* = *(Number of NPs taken up by cells/Number of NPs incubated with cells) ^∗^ 100%.* To obtain number of AuNP in each sample, total ppm determined by ICP-MS was divided to the mass of one AuNP. Total AuNPs number was divided to cells number as follow: number of AuNPs/n° of cells = (total mass of AuNPs by ICP-MS/mass of one AuNP)/n° of cells (where the *mass of one AuNP* = ρ *4/3 ρ^3^*π *with ρ* = *density of gold; r* = *radius of one AuNP).*

### Confocal Laser Scanning Microscopy (CLSM)

SKBR-3, cells were seeded on cover glasses with a density of 50.000 cells in a 24 well culture plate. After 24 h incubation, cells were treated with Trastuzumab free (2 μg/mL), 2.5Au@PT, 5Au@PT nanoparticles (2 μg/mL) and without any control for 24 h. Untreated cells were used as control. At the end of the culture time, the cells were washed with PBS, fixed with 4% (w/v) paraformaldehyde solution for 15 min, permeabilized with 0.1% Triton X-100, and blocked with Bovine Serum Albumin (BSA 3% in 1X PBS) for 1 hour at room temperature. Finally, cells were incubated with Alexa-Fluor-488–conjugated secondary antibodies anti-human (diluted 1:1000) for 45 min at room temperature (Invitrogen; excitation/emission maxima ∼ 495/519 nm). At the end of the incubation, the cells were washed in PBS, counterstained with a solution of Hoechst 33342 (2 μg/mL in 1X PBS; excitation/emission maxima ∼361/497 nm) to target the cellular nuclei, and then washed. Finally, samples were observed with a confocal fluorescence microscope (Leica TCS SPII Microsystems, Wetzlar, Germany). The AuNPs were visualized in reflection bands after the excitation at 545 nm.

### Ultrastructural Analysis

For the ultrastructural analysis, cells underwent a Silver enhancement made with Silver Enhancer Kit (Sigma-Aldrich) in order to increase the signal of colloidal gold particles. Subsequently, cells were fixed with 2.5% glutaraldehyde in PBS and a post-fixation with 1% osmium tetroxide in dH_2_O was performed to fix lipids. The samples were gradually dehydrated in acetone and embedded in epoxy resin. Thin sections of 70–80 nm were obtained with Reichert OM3 ultramicrotome and placed on formvar-carbon-coated nickel grids (300 Mesh). Finally, the grids were stained with uranyl and lead, using an unstained grid as control. All the samples were observed on a Zeiss EM900 electron microscope operating at 80 kV.

### Endocytosis Pathway Determination

Cells were seeded in 6-well culture plates at a density of 500000 cells/well, after 24 h incubated with different endocytosis inhibitors [2.5 mM amiloride (Sigma-Aldrich), 2.5 μg/mL chlorpromazine (Sigma-Aldrich) and 100 μM indomethacin (Sigma-Aldrich)] and then maintained for 1 h at 37°C with 5% of CO_2_-air. The concentrations and treatment times of each chemical inhibitors were optimized in a preliminary experiment to select the maximum non-toxic doses and treatment times. After 1 h incubation, the inhibitors were removed, and the cells exposed to 2 μg/mL AuNPs in complete medium for 24 h incubation. Then ICP-MS protocol was followed as described in the previous section.

### Western Blot Analysis

Cells were scraped from all the samples, including T, and lysed with ice-cold lysis buffer (1X RIPA buffer containing 1 mM and 1X protease inhibitor (Protease Inhibitor Tablets, SIGMA) for 30 min on ice. The lysates were then used for western blot analysis according literature protocol (Cristofaro et al., 2018). Primary antibodies anti-phosphorylated AKT and anti-AKT (diluted 1:500), anti-ERK and anti-phosphorylated ERK (diluted 1:1000), anti-BAX and anti-BCL-XL (diluted 1:500), anti-β-actin (diluted 1:500) and appropriate secondary antibodies HRP-conjugated were used. Detection was performed as described in the dot-blot section. Bands densitometry analysis was carried out with Image J software.

### Statistical Analysis

All statistical calculations were carried out using GraphPad Prism 5.0 (GraphPad Inc., San Diego, CA, United States). Statistical analysis was performed using Student’s unpaired *t*-test and through one-way variance analysis (ANOVA), followed by Bonferroni *post hoc*, for multiple comparisons (significance level of *p* ≤ 0.05).

## Results and Discussion

### Synthesis of AGMA1-SH

**FIGURE 2:**
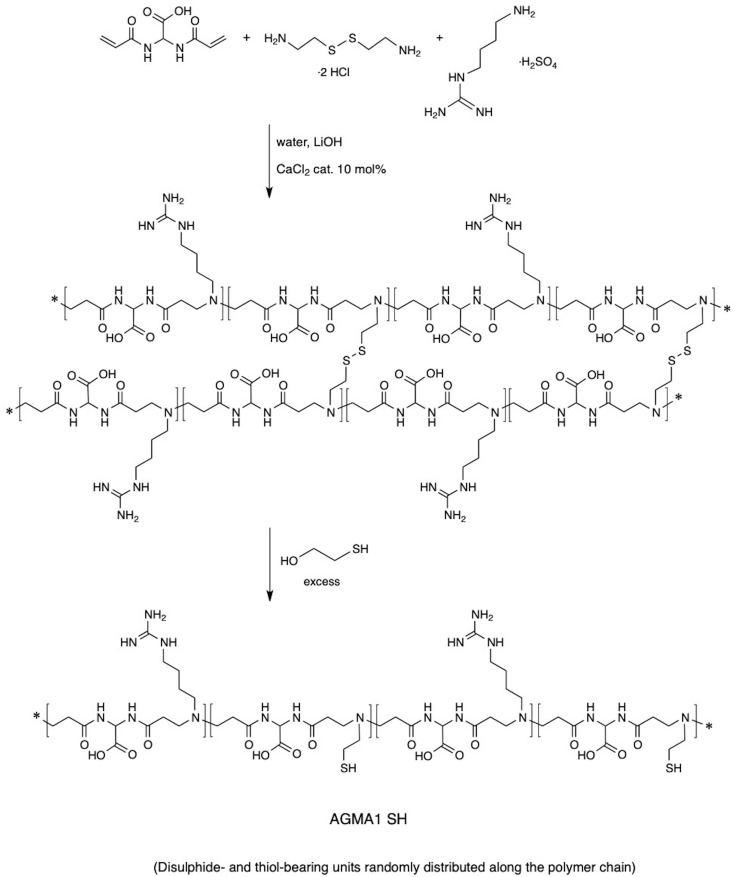
Synthesis of AGMA1-SH.

AGMA1-SH bearing 20% randomly distributed thiol-functionalized repeat units was synthesized following a two-step procedure previously reported for obtaining thiol-functionalized ISA23 (a cytobiocompatible, stealth-like PAA deriving from the polyaddition of 2,2-bisacrylamidoacetic acid with 2-methylpiperazine) (Donghi et al., 2009). According to this procedure (Figure 2), at 20% on a molar basis, cysteamine-deriving units were introduced in AGMA1 by substituting in the preparation recipe 10% cystamine for 20% agmatine. Cystamine, being a tetrafunctional monomer in Michael-type polyaddition acted as a crosslinker, and a soft hydrogel was obtained. Cystamine disulfide groups were subsequently reductively cleaved with excess 2-mercaptoethanol. The major portion of AGMA1-SH repeat units still bore 4-aminobutylguanidine pendants, as AGMA1, therefore it maintained its favorable cell-adhesive properties, but also contained cysteamine moieties capable of ensuring strong interactions with gold.

### Synthesis of Gold Nanoparticles Decorated With AGMA1-SH and/or Trastuzumab

Three differently sized AGMA1-SH-coated AuNPs decorated with Trastuzumab were synthesized according to a previously established green and robust route (Comotti et al., 2004). The aim was to assess how variously sized nanosystems could affect the biological activity, in particular endocytosis and apoptosis. In order to evaluate the role of each component inside the nanosystem, three differently sized AGMA1-SH-coated AuNPs and Trastuzumab-coated AuNPs were also prepared for comparison. Tuning gold nanoparticles diameter was expected to change anti-cancer ability of the nanosystem as a consequence of different surface to volume ratios which might affect the gold interaction with the polymer and drug, as well as their surroundings. On this regard, Bond and Thompson published a plot able to determine gold dispersion per nanoparticle (percentage of surface gold atoms with respect to gold atoms per nanoparticle) as a function of nanoparticle diameter (Bond and Thompson, 1999). Surface area and gold atoms number versus diameter can be determined as well (Supplementary Figure S1). Accordingly, the smaller the particles, the higher the fraction of atoms on the surface thus more gold sites are available for interaction. In particular, the three tailored AuNPs sizes (2.5, 3.5, and 5 nm) would lead to marked different gold dispersions of around 55, 35, and 25%, respectively. All the nanosystems were stable over time due to the mentioned Au-S soft-soft interaction, since sulfur is present either in AGMA1-SH or Trastuzumab (Della Pina et al., 2009). Briefly, the AuNP *core* (produced by chloroauric acid reduction, see below) was coated with the biocompatible polymer AGMA1-SH in order to prevent the nanosystem either from aggregation or ingestion by phagocytes (‘*stealth effect’*). The subsequent conjugation to an anticancer molecule (the antibody drug Trastuzumab) would enable gold nanovectors to recognize and inhibit only tumor cells. HAuCl_4_ aqueous solution served as a gold source, whose concentration was tuned to achieve the desired size of AuNPs and, hence, of the whole nanosystem. Proper amounts of AGMA1-SH were then added and Au^3+^ was instantly reduced to Au^0^ by NaBH_4_ addition without the presence of any conventional stabilizer (namely PVA, PVP or sodium citrate, often cytotoxic) due to the intrinsic protecting effect of the polymer. Shortly after, the polymer-coated AuNPs were decorated with the drug. Only in the case of 5Au@PT, 5Au@P, and 5Au@T samples, sodium borohydride was added prior to the polymer or antibody drug addition in order to allow gold nanoparticles to grow up to 5.0 nm. Both the polymer and the drug, in fact, were found to hinder gold nanoparticles growth when present before the reducing agent addition, probably due to their inhibiting effect against aggregation (Figure 1). More importantly, the innovative protocol introduces some advantages with respect to other synthetic routes commonly adopted (Rossi et al., 2016). First, the use of thiol-functionalized biocompatible PAAs allows to avoid conventional stabilizers, often cytotoxic, *via* Au-S bonds, which ensure long-term stability of the nanosystem without cytotoxicity. Second, the use of an instant reducing reagent (NaBH_4_), whose excess can be easily removed by dialysis, leads to homogeneously sized nanoparticles, simple to be tuned by HAuCl_4_ concentration.

### Physicochemical Characterization of Gold Nanoparticles Decorated With AGMA1-SH and/or Trastuzumab

**FIGURE 3:**
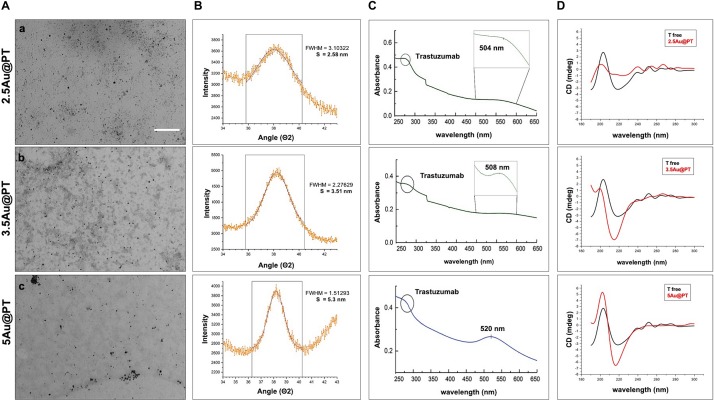
Characterization of Trastuzumab-conjugated gold nanoparticles with different sizes. **(A)** TEM images of 2.5 **(a)**, 3.5 **(b)**, and 5 **(c)** nm core diameter Au@PT nanoparticles (Scale bar = 200 nm), **(B)** XRPD, **(C)** UV-vis absorbance spectra (circle at 280 nm indicates the Trastuzumab peak), and **(D)** circular dichroism measurements of each Trastuzumab-conjugated gold nanoparticles families as function of core size.

Direct TEM observation of Au@PT complexes showed that all colloidal solutions contained monodispersed and spherical-shaped nanoparticles matching their designs (Figure 3A). As reported in the experimental section, the gold nanoparticle average diameter was determined by XRPD analyses using *Scherrer* equation. All colloidal samples were absorbed on XC72R carbon (1%Au/C) and the peak at *2*θ = 38.5°, typical of metallic gold, was considered. A long experimental work allowed to reach the expected theoretical values for most of the samples, especially when the polymer and the drug were both present (Figure 3B). Size evaluation of the whole colloidal nanosystem (gold – polymer – drug) required DLS technique. DLS measures the time-dependent fluctuations in the intensity of scattered light from a suspension of particles undergoing random *Brownian* motion. Analysis of these intensity fluctuations allows the determination of diffusion coefficients, which in turn yield the particle size through the Stokes-Einstein equation. Table 1 reports the hydrodynamic diameter average and Z-potential for all samples. Particles size and Z-potential are very important parameter to evaluate the stability of colloidal system (Sun et al., 2016). Absolute values of Z-potential above ± 30 mV are typically considered as an indicator of suspension stability against aggregation due to charge stabilization (i.e. the electrostatic repulsive forces are high enough to counteract aggregation) (Lowry et al., 2016). Meaningfully, Z-potential value can provide an explanation about the absorption mechanisms of drugs and biological ligands on nanoparticles surface. Likewise, it can be a key factor in determining the interaction with the cells. As listed in Table 1, all the AuNP spheres (with or without AGMA1-SH) showed a positive Z-potential value. However, some significant differences were observed. In absence of AGMA1-SH, the Z-potential values of Au@T nanoparticles decreased dramatically toward Z-values closed to neutrality when particle size increased, especially for the 5Au@T nanoparticles, which indicated a very low stability of the nanosystems. The reason can be attributed to the absence of the stabilizing effect of AGMA1-SH, that if present on the surface may impedes nanoparticle aggregation by steric hindrance to prevent surface interactions between AuNPs (Gao et al., 2012). After the addition of AGMA1-SH, the Z-potential value of the differently sized Au@P intensely increased. The most relevant increase was observed for 5Au@P (from 0.2 to 33.8 mW), confirming the impact of AGMA1-SH to prevent nanoparticles aggregation and deposition. After the Trastuzumab conjugation, a slight and not significant decrease on Z-values was assessed in 2.5 and 3.5Au@PT suspensions than 2.5 and 5Au@P counterparts (24.2 to 18.4 mV for 2.5Au@PT and 23.0 to 21 mW for 3.5Au@PT, respectively). With value higher than 30.0 mW, 5Au@P and 5Au@PT represent the most stable nanoparticles, underling a possible effect due to the size of the nanospheres. Numerous strategies explored the attachment of a targeting ligand (i.e. sugars and peptides) through S-Au bonds (Bertrand et al., 2014; Doulain et al., 2015). Although some drawbacks, particular promising are those exploiting these bonds for the functionalization with molecules for biosensing, anticancer drug delivery (i.e. antibodies), and bioimaging (Ghosh et al., 2008; Gautier and Cisnetti, 2012). Recently, Matos et al. (2018) used an engineered Trastuzumab presenting an additional free cysteine per light chain, obtaining a stable and homogeneous gold thiol-linked Thiomab conjugate with unmodified anticancer activities. Sulfur is a component of Trastuzumab itself (C_6470_H_10012_N_1726_O_2013_S_42_, hence 42 Sulfur atoms per molecule). Hence, it is reasonable to speculate that its free thiol groups may contribute on the stability enhancement of AGMA1-SH-coated gold nanoparticles interacting directly with the gold surface. The UV-vis spectra displayed a plasmon resonance band (PRB) in the strict range 498–504 nm for the smallest samples (2.5Au@PT, 2.5Au@P, and 2.5Au@T), 508–515 nm for the intermediate-sized samples (3.5Au@PT, 3.5Au@P, and 3.5Au@T) and 517–525 nm for the largest ones (5Au@PT, 5Au@P, and 5Au@T) (Figure 3C and Supplementary Figure S2). The location of PRB peak strongly depends on the size of the metal nanoparticles, as well as on the surrounding medium and its dielectric constant. In particular, the larger the nanoparticles, the higher the wavelength. Hence, the detected PRB values are coherent with the expected theoretical trend (Figure 3C).

**TABLE 1:** Size distribution of hydrodynamic diameters (d_h_) and Z-potential as determined by DLS.

	Number size	
Sample	distribution [nm]	Z potential [mV]
AGMA1SH	1.3 (97.6%) ± 0.2;	
	3.2 (2.4%) ± 0.7	
Trastuzumab	9.3 (100%) ± 2	
2.5Au@PT	6.0 (100%) ± 1	18.4 (85.1%) ± 2.7; 3.2 (12.9%) ± 1.7
3.5Au@PT	9.5 (100%) ± 3	21.0 (86.2%) ± 7.2; 3.8 (13.8%) ± 1.3
5Au@PT	27.0 (100%) ± 6	32.6 (100%) ± 9.4
2.5Au@P	5.4 (100%) ± 1	24.2 (69.6%) ± 2.6; 6.7 (27.3%) ± 3.3
3.5Au@P	14.8 (100%) ± 5	23.0 (73.3%) ± 3.0; −2.8 (26.7%) ± 1.6
5Au@P	22.1 (100%) ± 7	33.8 (100%) ± 7.6
2.5Au@T	19.9 (100%) ± 6	7.4 (100%) ± 3.5
3.5Au@T	63.1 (100%) ± 21	13.6 (100%) ± 7.5
5Au@T	77.0 (100%) ± 29	0.2 (100%) ± 0.1
		

In addition to the physicochemical characteristics of all nanoparticle’s formulations tested, the efficiency of the conjugation reaction was also determined (Table 2 and Supplementary Table S2). Trastuzumab was conjugated to the Au@P with an efficiency of ∼50–70%. In general, the percentage of conjugation efficiency augmented by increasing the gold nanoparticles sizes. It was approximately of 50.2 ± 3.9% for 2.5Au@PT, 60.0 ± 6.3% for 3.5Au@PT and 69.4 ± 7.8% for 5Au@PT, respectively, denoting that the size of gold nanoparticles intensely impacts the binding with the targeting molecules. The relative amount of T (μg) per nanoparticles (μg) are also reported (Table 2). Beyond to visualize Trastuzumab on the nanoparticles as spots on the nitrocellulose membrane, dot blot assay confirmed the indirect values obtained by BCA analysis (Supplementary Table S3 and Supplementary Figure S3). Size-related effect was also recorded in the Trastuzumab-functionalized nanoparticles without AGMA1-SH. It is worth noting that, in all these nanoparticles, the amount of Trastuzumab per nanoparticles was lower than AGMA1-SH-coated gold nanoparticles (Supplementary Table S2). Gao et al. (2012) documented that a thiol-ending polyethylene glycol (PEG-SH), significantly improved to colloidal stability of gold nanoparticles in aqueous solution, facilitating their conjugation with epidermal growth factor receptor antibodies. Hence, presumably the AGMA1-SH presence, stabilizing the gold nanoparticles, may be crucial for the establishment of a successful interaction with the antibody (Gao et al., 2012). In nanobiotechnology applications, curvature of nanoparticles has a significant effect on protein structure and activities, although little is exactly known about molecular level mechanism. Likewise, hydration layer above the nanoparticles surface, the ionic strength of buffer solution and stabilizing polymers concentration can be critical factors for protein interaction with nanoparticles (Iijima and Kamiya, 2009; Kushida et al., 2014; Yu and Zhou, 2016; Lin et al., 2017). Kushida et al. (2014) showed that the interaction of the coagulation proteins with silica nanoparticles decreases with the decreasing NP size (higher curvature). Vertegel et al. (2004) observed a greater loss of lysozyme conformation adsorbed onto larger silica nanoparticles under otherwise similar conditions. About the gold nanoparticles, the effects of curvature on ligand interaction remains a complicate issue (Villarreal et al., 2017). Villarreal et al. (2017) CD spectroscopy method is commonly used to determine protein structure and its interaction with other molecules. CD spectroscopy confirmed the Trastuzumab conjugation to the gold nanoparticles (Figure 3D and Supplementary Figure S4), but also suggested a correlation between Trastuzumab-conjugated secondary structure maintenance and the particle hydrodynamic radius (R). Consistently with literature findings (Jiang et al., 2005, 2008; Worrall et al., 2006; Saptarshi et al., 2013), the correct folding increased with R growing from 2.5Au@PT to 5Au@PT (Figure 3D). Only the largest nanoparticles (5Au@PT) displayed CD spectra superimposable to the unconjugated antibody, indicating that the nanoparticles are a highly efficient and selective tool for HER-2 receptor. Previous studies hypothesized that the higher surface curvature of smaller nanoparticles may restrict the relative orientation between antibodies, resulting in a certain degree of conformational rigidity, that while blocking the correct protein folding, it impaired their docking receptor surface (Jiang et al., 2008). By contrast, for conjugates built on larger sizes, the antibody, once absorbed on nanoparticles surface, extended itself into the surrounding medium in a conformation allegedly better suited to interact with its receptors (Ghitescu and Bendayan, 1990; Jiang et al., 2008; Saptarshi et al., 2013). Concerning the *in vitro* release of Trastuzumab, each type of Au@PT presented a low released amount in MilliQ water up to 15 days of investigation (5.3 ± 0.7%, 4.4 ± 0.5%, and 3.5 ± 0.6% for 2.5, 3.5, and 5Au@PT, respectively) (Supplementary Figure S5). Interestingly, analyses up to 12 months showed that 5Au@PT complex was the most stable of storage at refrigerated conditions, without any aggregate’s formation and with an approximately release of 20 ± 0.3%. By contrast, both 2.5 and 3.5Au@PT displayed a tendency to precipitate after few months from their synthesis. Challenging and crucial aspect of nanoparticles characterization is the measurement of their stability under condition resembling *in vitro* or *in vivo* environment. Numerous studies revealed that the proteins, salts, and antibiotics present in the different culture media can interact with AuNPs, determining radical changes in their size by forming aggregates and reducing their stability (Moore et al., 2015). Notably, all these components can confer the particles with a new biological identity and then dramatically alter their interaction with the cells (Gebauer et al., 2012). Therefore, it has been becoming increasingly evident that the nanoparticles toxicity and activity assessed *in vitro* entails the mixing nanoparticles of interest with the biological media for enhancing the comprehension of their interaction with the biological systems. Hence, to mimic the cell bioenvironment prior to cells treatment, freshly synthesized batches of Au@PT nanoparticles were re-suspended in the cell culture media (containing 10% of serum) proper for each cell lines used for biological investigation. Hydrodynamic changes were assessed by DLS analysis at 37°C after 4 h of incubation. As shown in Table 3 after serum incubation, the hydrodynamic diameter of the three types of Au@PTs (in particular for the smaller ones) increased suggesting the serum proteins adsorption on nanoparticle surface, that forming a “corona.” Although with discrepancies in the results, numerous studies on different types of nanoparticles and on various cell lines highlighted the significance of protein corona (Hajipour et al., 2015). They profoundly influence the aggregation of nanoparticles and their cellular uptake (Dominguez-Medina et al., 2016). Also, it has been demonstrated that the protein corona composition affects the internalization of gold nanoparticle coated with different polymers (Walkey et al., 2014). Recently, it was found that the “*stealth effect*” of poly(ethylene glycol) may be explained by the presence of specific proteins in their protein corona (Schöttler et al., 2016). As summarized by Moore et al. (2015) the type of basal medium can dictate the size and stability of nanoparticles. Results from Strojan et al. (2017) demonstrated that the medium in which silica nanoparticles were dispersed, had significantly affected nanoparticles protein corona composition, suggesting an important implication on nanoparticles potential biological effects. Ji et al. (2010) have found that titanium dioxide (TiO_2_) nanoparticles increased their hydrodynamic in the presence of cell culture media without serum. By contrast, the addition of bovine serum protein stabilized nanoparticles, as there was only a little change in size following the addition of proteins (Spadavecchia et al., 2016). Moreover, as previously reviewed, different surface modifications (i.e. by using biocompatible polymers coating) can be used for lessening the protein corona formation then improving the stability of nanoparticles in liquid media (Iijima and Kamiya, 2009). Based on and in agreement with literature findings (Colombo et al., 2016), we inferred that the small increase observed on 5Au@PT nanoparticle size, in all complete media tested, might due to the higher surface functionalization with T, which decreased the extent of serum protein adsorption. By contrast, the lower amount of antibody per nanoparticle on 2.5Au@PT and 3.5Au@PT may favor protein absorption then resulting in an increase of hydrodynamic diameter. Overall, protein adsorption may be hindered by the functionalization of gold nanoparticles with AGMA1-SH and Trastuzumab, but it cannot be totally inhibited, and a certain amount of serum proteins do adsorb on the NP surfaces, irrespective to the presence of polymers coating, justifying then the small increase of the hydrodynamic size.

**TABLE 2:** Conjugation efficiency of AGMA1-SH-AuNPs.

Samples	Conjugation efficiency%^a^	μg of Trastuzumab per 20 μg of nanoparticles^b^
2.5Au@PT	50.2 ± 3.9	10 ± 0.8
3.5Au@PT	60.0 ± 6.3	11.9 ± 1.3
5Au@PT	69.4 ± 7.8	13.9 ± 1.6

*^*a*^Conjugation efficiency% = (1 − ([Trastuzumab in the supernatant]/[Trastuzumab added in the conjugation reaction])) × 100. ^*b*^μg of Trastuzumab per 20 μg nanoparticles = Trastuzumab added in the conjugation reaction − Trastuzumab in the supernatant.*

**TABLE 3:** Hydrodynamic diameter (nm) of Trastuzumab-AGMA1-SH-gold nanoparticles dispersed in complete cell culture media at two different time points.

	2.5Au@PT		3.5Au@PT		5Au@PT	
			
Media	0 (h)	4 (h)	0 (h)	4 (h)	0 (h)	4 (h)
McCoy’s + 10% FBS	7.3 ± 1	17.5 ± 2	10.9 ± 1	18.7 ± 1	20.1 ± 1	27.0 ± 1
MEM eagles + 10% FBS	9.1 ± 1	16.5 ± 2	8.3 ± 3	16.6 ± 1	23.3 ± 1	32.3 ± 1
DMEM + 10% BCS	9.7 ± 2	21.5 ± 2	13.0 ± 1	30.9 ± 4	21.9 ± 1	23.7 ± 3

*Mean value and standard deviation of three measurements per condition are reported.*

It is worth emphasizing that the NPs characterization in standard conditions for the *in vitro* biological experiments, i.e. in serum-containing cell culture medium, is still a questioned issue. Indeed, additional determining factors can regulate the nanoparticles amount reaching the cell monolayer and the type of interaction with cells. Cell types and its protein component, including the protein secreted by cells, contribute to affect the deposition of the nanoparticles in the cell-well (Halamoda-Kenzaoui et al., 2015). Recently, it was observed that nanoparticle administration method *in vitro* cell culture could alter particle-cell interaction (i.e. cellular adsorption and uptake) (Moore et al., 2019). All the factors make hard to envisage the exact behavior of the nanoparticles once in contact with the cells and should to be taken in account during the biological experiments. Whilst bearing in mind that accurate experiments are required, overall characterization proved the efficacy of the use of AGMA1-SH to produce stable gold nanoparticles. Consequently, their anticancer activities were subsequent evaluated by *in vitro* cell models as follows described.

### *In vitro* Cytotoxicity and Uptake: Evaluation in Breast Cancer and No-Neoplastic Cell Lines

*In vitro* and *in vivo* successful achievements employing delivery synthetic nanoplatforms need targeting molecules to recognize tumor cells specifically, without affecting the surrounding healthy cells.

**FIGURE 4:**
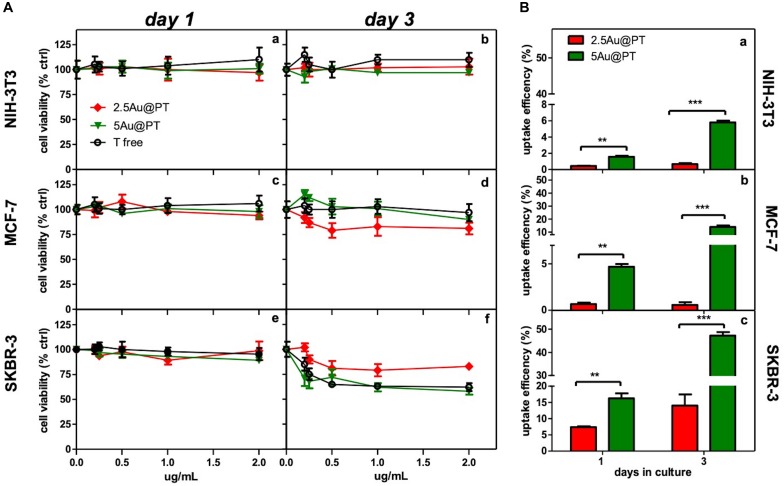
Cytotoxicity and internalization of Trastuzumab-conjugated gold nanoparticles differently sized. **(A)** Dose- and time-dependence cell viability of different cell lines cultured with free Trastuzumab, 2.5Au@PT and 5@AuPT. SKBR-3 **(e,f)** and MCF-7 cells **(c,d)** were incubated with 2.5Au@PT or 5Au@PT suspensions with concentration in the range of 0–2 μg/mL. Similarly, the experiment was performed in NIH-3T3 fibroblasts cell line **(a,b)** as negative control. After 1 and 3 days of incubation, cell viability was expressed as percentage of viability determined in the absence of nanoparticles (set at 100%). **(B)** Uptake efficiency of 2.5Au@PT and 5Au@PT (2 μg/mL) after 1 and 3 days of incubation were determined by ICP-MS in all the indicated cell lines (**a**, NIH-3T3; **b**, MCF-7; **c**, SKBR-3) and calculated as described in *Experimental Section* (mean values ± standard deviations; **p < 0.01 and ***p < 0.001).

Aiming to assess the activities of Au@P functionalized with Trastuzumab, SKBR-3, human breast cancer cell line was selected as target cell model. This cell line overexpresses HER2, itself the target of Trastuzumab, and has been used extensively as an *in vitro* model for evaluating the anticancer targeting of nanoparticles as tool to enhance the selectivity and specificity of therapies against cancer cells (Kim et al., 2011; Martínez-Jothar et al., 2019; Niza et al., 2019). Moreover, to confirm the selective effect of Au@PT toward the HER2-receptor, others two cell lines were included for comparative analysis: MCF-7, a human breast cancer expressing low HER-2 as control, and the murine fibroblasts cell line, NIH-3T3, as health cell model. Both are commonly used in experimental studies for assessing the selectivity of anticancer treatments and the nanomaterials toxicity (Xu et al., 2005; Holliday and Speirs, 2011; Shirshahi et al., 2013; Merli et al., 2018; Cruz and Kayser, 2019). All *in vitro* experiments were conducted treating all cell types (1 × 10^4^ viable cells/well) with increasing concentrations (from 0 to 2 μg/mL) of each Au@PT size and free-Trastuzumab. The concentration range was fixed by determining the “no toxic” dose by incubating the same number of cells with increasing concentrations of the unfunctionalized Au@P counterparts (Supplementary Figure S6). A low cytotoxicity was observed up to 2 μg/mL, then the concentration range upper limit was set at this value. As reported in Table 2, 2 μg/mL of 2.5Au@PT, 3.5Au@PT, and 5Au@PT contain approximately 1–1.4 μg of Trastuzumab, which was the concentration within the effective doses against breast cancer cells (Suarez et al., 2013). 2.5Au@PT, 3.5Au@PT, and 5Au@PT cytotoxicity was tested by measuring cell viability using MTT after incubation with Au@PT. 2.5Au@PT and 5Au@PT were particularly effective in impairing the SKBR-3 viability, whereas they had low or no toxicity on MCF-7 and NIH-3T3 respectively (Figure 4A). Interestingly, cells exposed to Au@PT underwent viability decrease after 3 days of incubation (around 50 and 20% with 5Au@PT and 2.5Au@PT respectively). It could be argued that the difference in cells response is due to the changes observed in the secondary structure of the antibody, which was able to affect the binding capacity of Trastuzumab-AuNPs with HER-2 receptors. 3.5Au@PT was comparable to that of 2.5Au@PT treated cells, nearby 25% at 2 μg/mL (data not shown). Targeting molecule may reduce their targeting capacity and activities as protein corona adsorption (Mirshafiee et al., 2013). Also surface rigidity, ligand density affects nanoparticles effect on cell (Gong et al., 2015). Consequentially, it is conceivable that lower activity detected after the treatment with 2.5Au@PT, the ones showing the highest increase of hydrodynamic size following the incubation with the complete cell media, may be related to protein corona absorption. However, other explanations cannot be excluded. Pan et al. (2007) have demonstrated that the cytotoxicity of modified gold nanoparticles is dependent on the size and not on a particular ligand attached to them. In addition, some researchers showed that because metallic, these nanoparticles have an effect on cell membrane integrity, and their size and charge both affect significantly cell viability (Woźniak et al., 2017). Similarly, cellular uptake of gold nanoparticles was influenced by many factors including size, charge, coating, shape, incubation time and cell types (Dreaden et al., 2012; Panariti et al., 2012; Yue et al., 2017). Chithrani et al. (2006) observed that size and shape strongly influenced cellular uptake of citric acid ligand-modified Au nanospheres, and others reported a clear interrelationship between nanoparticles surface functionality an uptake (Jiang et al., 2015). Meanwhile, the adhesion of nanoparticles onto cell membrane can be reduced because of protein corona formation around nanoparticles surfaces, which in turn inhibit their cellular uptake. As reported by Cheng et al. (2015) protein from serum, modulating biomolecular corona profile, changed the AuNPs internalization in a size- and cell type-dependent manner. Aiming to explore the size contribution in Au@PT uptaking, the succeeding experiments were performed using nanoparticles differing for about fourfold in hydrodynamic radius, 2.5Au@PT and 5Au@PT. Others have previously underlined that an equilibrium between multivalent crosslinking of membrane receptors and membrane wrapping involved in receptor-mediated endocytosis exists (Jiang et al., 2008; Yue et al., 2017). Jiang et al. (2008) showed that extremely small (2 nm) or large Trastuzumab-conjugated nanoparticles (>50 nm) would both produce an inefficient uptake. Aoyama et al. (2003) and Osaki et al. (2004) investigated size effects and receptor activation, concluding that receptor-mediated endocytosis is firmly size-dependent with an optimal size equal to ≈25 nm. Others reported a theoretical model based on size and receptor-mediated endocytosis that gives an optimal radius ≈27–30 nm for spherical particles (Gao et al., 2005). Besides, Wang et al. (2010) using a 3D image process identified the AuNPs distribution, revealing that the optimal size is 45 nm for cells uptake. The results here described seem to show a broader agreement with previous experimental observations. Indeed, as compared to 2.5Au@PT (d_h_ ∼ 6 nm), a significant enhancement in the uptake of 5Au@PT (d_h_ ∼ 22 nm) was detected in HER-2 overexpressing human breast cancer SKBR-3 cells (Figure 4B). Nonetheless, the internalization of both 2.5Au@PT and 5Au@PT was highly dependent on time (Figure 4B). Large nanoparticles aggregates are not suitable for cellular interaction and uptakes. Using SEM-BSE mode, it was easily appreciated smaller 5Au@PT cluster coating homogenously SKBR-3 cells surface, whereas a closely 2.5Au@PT were packed into bigger aggregates (Supplementary Figure S7), suggesting the difficult capture of the latter by cells. In addition, uptaking studies using unconjugated-Au@P confirmed the role played by the size, with the most efficient uptake occurring with the largest nanoparticles (5Au@P; d_h_ ∼ 22 nm) (Supplementary Figure S8).

The accumulation of Au@P and Au@PT inside the cells could be regulated also by the exocytosis process. It was found that Au NPs were exocytosed in a linear manner in size, with smaller AuNPs exhibiting a faster exocytosis rate and percent (after 8 h of incubation, 4 nm-AuNPs were exocytosed while > 90% of 74 nm remains into the cells) (Chithrani and Chan, 2007). Equally, the thermodynamic process could be the driving force for wrapping. In general, smaller particles must be clustered together to create enough driving for uptake, showing an uptake amount smaller than 50 nm AuNPs (Gong et al., 2015). Recently, Cruz E and Kayser V have found that Trastuzumab-attachment increased nanoparticles cellular uptake in HER2 amplified cell lines selectively (Cruz and Kayser, 2019). Although higher uptake is required in delivery applications, internalization must be specific to the targeted tumor cells. In agreement, the observation that Au@PT internalization was higher in HER2 overexpressing cell line (SKBR-3) than in both MCF-7 and NIH-3T3, it was a further evidence that cellular uptake increased through the HER2 receptor crosslinking Trastuzumab-activated (Figure 4B). Likely, the low percentage of uptakes detected in MCF-7 and NIH-3T3 was due to passive internalization (Figure 4B) (Zhao and Stenzel, 2018). Later, in line with previous studies(van der Meel et al., 2012; Martínez-Jothar et al., 2019), SKBR-3 cell line showed a significantly increased uptake for Trastuzumab-conjugated AuNPs as compared to non-targeted nanoparticles (50% vs. 10%, respectively after 3 days of incubation); these data further proved that the uptake of antibody-targeted nanocarriers is truly mediated by specific ligand-receptor interactions.

### HER Targeting: Evaluation of Morphology, Viability and Intracellular Changes After Au@PT Treatment

**FIGURE 5:**
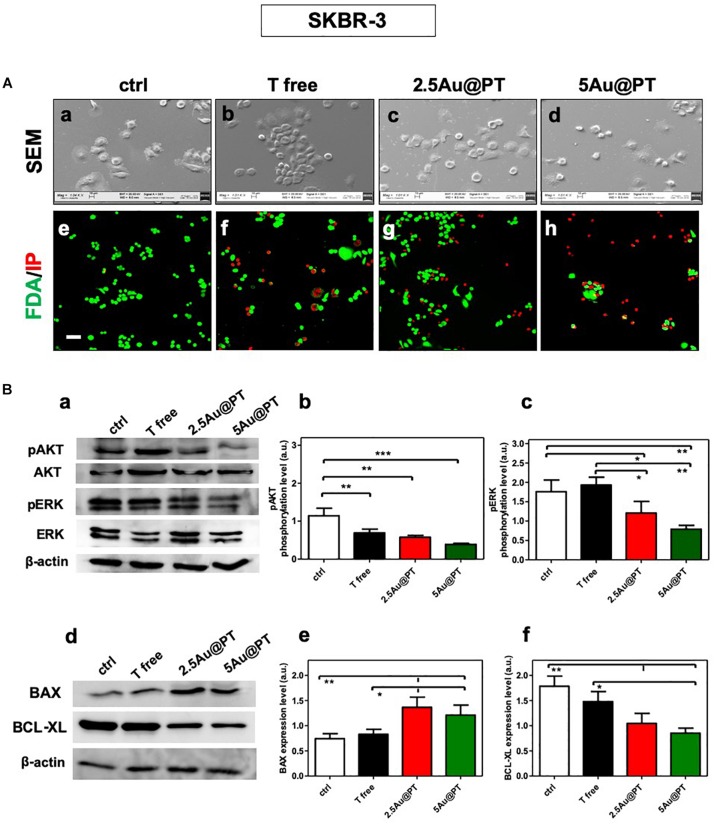
**(A)** Cell morphology assessed by scanning electron microscopy in ctrl **(a)**, with Trastuzumab **(b)**, 2.5Au@PT **(c)** and 5Au@PT **(d)**, respectively, after 3 days of incubation. Scale bars = 100 μm and mag: 1000×. After 3 days of culture, all experimental groups [ctr **(e)**, T free **(f)**, 2.5Au@PT **(g)**, and 5Au@PT **(h)**] were treated with fluorescein diacetate (green cells alive) and propidium iodide (red cells dead) 20× magnification, the scale bar represents 50 μm. **(B)** Western blotting analysis of survival **(a,b)**, proliferative **(a,c)**, pro **(d,e)**, and anti-apoptotic **(d,f)** proteins extracted from SKBR-3 after 24 h incubation with the indicated treatments. The activation level of AKT or ERK is presented as a ratio between the phosphorylated and total AKT or ERK protein after normalization to β-actin housekeeping protein signal. Bar graphs show BAX and BCL-XL expression level obtained normalizing β-actin housekeeping protein signal. Statistical significance values are indicated as ***p < 0.001, **p < 0.01, and *p < 0.05.

The correct binding of a ligand molecule with cell surface receptors intimately controls the cell function and fate. Many works showed that the physical parameters of nanoparticles (i.e. size) strongly affect ligand binding and the subsequent activation of its membrane receptors (Chithrani et al., 2006; Brzóska et al., 2018). In line with quantitative results, FDA assays showed the highest number of death cells (red signal) in cultures exposed with 5Au@PT suspension (Figure 5A). SEM images clearly highlighted this important difference, displaying more spherical cells in shape with noticeable abnormal morphology in 5Au@PT-treated samples in comparison with control, free Trastuzumab or 2.5Au@PT (Figure 5A). The binding of the ligand to its surface receptors allows the formation of multivalent HER-crosslinking surface ErbB2 receptors, affecting intracellular signaling, consequently inhibiting downstream pathways involved in cell survival, proliferation, and metastasis (Klapper et al., 2000).

The strategy of combining the properties of AuNPs with anti-cancer molecules could be the future of cancer nanomedicine (Sun et al., 2018). Chattopadhyay et al. (2010) showed that Trastuzumab-PEG-AuNPs (30 nm) in combination with 300 kVp X-rays enhanced DNA double strand breaks (DSBs) in SKBR-3 cells. Mechanistic studies performed by Vemuri et al. (2019) revealed that the functionalization of naturally derived phytochemicals, such as Quercetin and Paclitaxel, to AuNPs were significantly effective in inhibiting cell proliferation, apoptosis, angiogenesis, colony formation and spheroid formation. AuNPs principally trigger apoptosis through intrinsic pathways, including mitochondria- and ER-related pathways. Green synthesized of AuNPs (10 – 42 nm) can induce the apoptosis activating the caspase-3 and 9 in human cervical cancer cells (Baharara et al., 2016) and the citrate-coated AuNPs (8 nm) promoted apoptosis eliciting BAX translocation and cytochrome c release in human liver (HL7702) cells (Gao et al., 2011). In agreement with literature findings (Sun et al., 2018; Vemuri et al., 2019), Au@PT cytotoxic effects elicited significant changes in survival and proliferation (AKT/PI3K and ERK pathways, respectively) as well as apoptotic process (pro-apoptotic BAX, anti-apoptotic BCL-xL, respectively) (Figure 5B). AKT and ERK activation levels were significantly reduced in the treatment with 2.5Au@PT and 5Au@PT than with control (Figures 5Ba–c), with a slight decrease after the incubation with 5Au@PT. Additionally, both 2.5Au@PT and 5Au@PT treatments led to a substantial increase in the expression of BAX with concomitant decrease in expression of BCL-xL in comparison with free Trastuzumab and control (Figures 5Bd–f). As mentioned, the most marked changes were observed after 5Au@PT incubation, still proving the size-effect dependence. Overall, the results showed that Trastuzumab-AGMA1-SH-gold AuNPs conjugates retain great antimitotic and anti-apoptotic effectiveness after the conjugation to Au@PT, which could improve the cancer therapeutic effects of Trastuzumab itself.

### HER-2 Targeting: Evaluation of Internalization by Confocal Laser and Transmission Electron Microscopies

**FIGURE 6:**
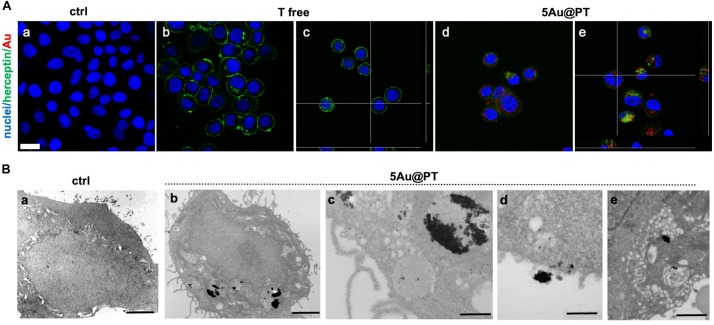
Internalization of 5Au@PT in breast cancer target cells, SKBR-3. **(A)** CLSM images of cancer cells incubated for 3 days in absence of 5Au@PT and Trastuzumab **(a)**, with free Trastuzumab **(b,c)**, and 5Au@PT **(d,e)**. Both Au@PTs were visualized in reflection bands after the excitation at 545 nm and stained in red false color. Trastuzumab was visualized by anti-human secondary antibody-488 Alexa Fluor. Nuclei were stained with Hoechst 33342 (blue). Scale bar: 20 μm. **(B)** TEM samples stained with uranyl and lead for cell morphology: **(a)** ctrl cells; **(b)** cells treated with 5Au@PT; uptake of large clusters inside vesicles is clearly visible after silver enhancement; **(c)** detail of a cell vesicle containing 5Au@PT; **(d,e)** high magnification of 5Au@PT putative uptake nearby the cell membrane; scale bars: 2 μm, 2 μm, 500 nm, 500 nm, and 500 nm respectively.

As a drug delivery platform, an efficient cell uptake is important to ensure sufficient therapeutic outcomes (Carnovale et al., 2016). Some studies suggest that Trastuzumab can induce a rapid internalization of HER-2 (Rubin and Yarden, 2001; Chattopadhyay et al., 2010; Han et al., 2014), a mechanism that could facilitate 5Au@PT uptaking by SKBR-3 cells. Co-localization of HER-2 and gold nanoparticles were then studied (Figure 6). In cells treated with free Trastuzumab, the antibody staining (in green) was predominant in cell membrane because of its interaction with HER-2 receptors (Figures 6Ab,c). On the other hand, cancer cells incubated with 5Au@PT exhibited a reduced Trastuzumab staining at the cell membrane (Figures 6Ad,e) but increased inside the cells and co-localized with 5Au@PTs as revealed by the scattering signals of gold (in red) (Figures 6Ad,e).

These data, besides proving the powerful and selective ability of Trastuzumab to promote the transport of 5Au@PT into HER-2 overexpressing cells, also suggest that receptors undergo endocytosis process when bound to Au@PT of a specific size range.

Transmission electron microscopy provides an excellent tool for in-depth biological sample analysis at the cellular and organ level. In the present work, it confirmed SKBR-3 cellular internalization of 5Au@PTs (Figure 6B). In (c) at higher magnification, the distribution of the NPs in close proximity of the vesicle’s membrane seems to evidence the binding between the drug and its receptor: a cluster of 5Au@PT was approaching the cell membrane (d) and was trapped inside vesicles of ranging sizes (e). The electron dense material appears to have a low resolution due to the silver enhancement procedure. However, a series of control experiments in absence of silver, osmium, uranyl and lead ruled out the possibility of artifacts as shown in Supplementary Figure S9. Generally, AuNPs were localized within early endosomes and lysosomes (Boyoglu et al., 2013; Xie et al., 2017). Therefore, it is presumable the vesicles structures visualized by TEM were the result of the endosome/lysosome trafficking induced by Trastuzumab binding to its membrane receptor. Some of these structures may be also autophagosome. Previously, it was observed gold nanoparticles induces autophagosome accumulation in accordance with size-dependent nanoparticle uptake and lysosome impairment (Ma et al., 2011). A high-resolution technique may help to clarify the full pathway of Au@PT intake and leading to a precise subcellular localization.

### HER-2 Targeting: Molecular Mechanisms Underlying the 5Au@PT Capture

**FIGURE 7:**
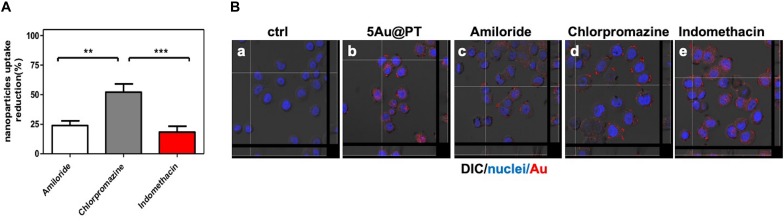
Cellular uptake mechanism of 5Au@PT quantified with ICP-MS **(A)** and observed by CLSM **(B)**. In both experiments, uptakes of 5Au@PT were analyzed in presence of specific endocytosis inhibitors as described in *Experimental Section* (2.5 mM Amiloride; 2.5 μg/mL Chlorpromazine; 100 μM Indomethacin). **(A)** ICP-MS data are expressed as nanoparticles reduction (%). Statistical significance values are indicated as ***p < 0.001, **p < 0.01. **(B)** CLSM-DIC (differential interference contrast) mode of cells untreated **(a)**, exposed to 5Au@PT **(b)**, and incubated with different endocytosis inhibitors (**c**, 2.5 mM amiloride; **d**, 2.5 mg/mL chlorpromazine; **e**, 100 mM indomethacin) for 1 h before the incubation with 5Au@PT for 24 h. Orthogonal view of images stacks is shown. Scattering of Au in red (false color) nuclei in blue. The focus of cells was adjusted using the differential inference contrast as a reference.

In conclusion, to underscore the sensitive endocytic route responsible for 5Au@PT internalization by target cells, three different endocytosis pathways inhibitors were employed (Figure 7): amiloride for micropinocytosis inhibition, chloropromazine for clathrin-mediated endocytosis (receptor dependent mechanism) and indomethacin for caveolae-mediated endocytosis inhibition. The uptake of 5Au@PT was dramatically affected by chloropromazine treatment (around 50% of reduction), whereas the other two inhibitors did not substantially modify the delivery into the cells (Figure 7A). Cross-section analysis confirmed the aforementioned results, revealing a strong scattering distribution along the cellular surfaces due to inhibition treatment (Figure 7B). Receptor-mediated clathrin carries out the uptake of specific macromolecules (ligands) or ligand-coated NPs following their binding to receptors on the surface of cell membrane, promoting the invagination of the plasma membrane to form area with a higher receptor concentration (Zhao and Stenzel, 2018).This type of mechanism has been widely used strategy for the drug delivery by nanocarriers (i.e. nanogel, liposomes). Saporin-loaded nanobody-targeted polymeric nanoparticles induced HER2 clustering, and promoting its internalization causes an efficient NPs receptor-mediated endocytosis with subsequent intracellular delivery of its cytotoxic cargo (saporin) (Martínez-Jothar et al., 2019). As regard AuNPs, Trastuzumab-functionalized AuNPs mostly underwent endocytosis in breast cancer cells largely through a receptor-facilitated mechanism (Chattopadhyay et al., 2010; Han et al., 2014; Rathinaraj et al., 2015; Sun et al., 2018). Han et al. (2014) found that quantum dots-HER bound specifically to SKBR-3 membrane, inducing their internalization via a receptor-mediated mechanism. In accordance with the aforementioned findings, the present results showed that the most probable mechanism for 5Au@PT nanoparticle endocytosis in breast cancer cells (SKBR-3) was receptor-dependent, suggesting that the AuNPs-mediated delivery of monoclonal antibody toward its specific cell membrane receptor was achieved efficiently.

In summary, the conjugation of the 5Au@Ps with Trastuzumab allowed their specific binding to HER-2-overexpressing cells, and through a receptor-mediated internalization process, may trigger their deposition into the cytoplasm, where gold nanoparticles, in combination with free Trastuzumab released into the cells, likely contribute to breast cancer cell death.

## Conclusion

The synthesis and the functionalization of gold-nanoparticles with cancer-specific biomolecules may represent a winner strategy for a selective and targeted tumor-phototherapy. Currently, a considerable number of efforts have been taken in order to obtain gold-nanoparticles by green and safe method. In this work, extra-small gold nanospheres stabilized with thiol-functionalized AGMA1 and Trastuzumab were for the first time synthesized and tested *in vitro* as nanovectors for breast cancer targeted drug delivery. The synthetic method followed an innovative protocol introducing significant advantages with respect to other routes presently adopted. *In primis*, tailoring thiol-functionalized biocompatible PAAs avoided the use of conventional stabilizers, often cytotoxic, *via* Au-S soft-soft interactions, which ensured long-term stability of the hybrid nanomaterial without any cytotoxicity. Importantly, sulfur is even a component of Trastuzumab itself. This represented an intrinsic, powerful tool for further stabilizing the composite nanosystem. Moreover, the employment of an instant reducing reagent (NaBH_4_) guaranteed homogeneously sized and tailored ‘on demand’ gold nanoparticles by properly tuning HAuCl_4_ concentration. The cytotoxicity and cellular uptake behaviors have been investigated in no-neoplastic cells (NIH-3T3) and in two breast cancer cell lines (MCF-7 and SKBR-3) by MTT assay and inductively coupled plasma mass spectrometry (ICP-MS) respectively. It was found that 5Au@PT with the highest hydrodynamic diameter and positive charge showed much higher cell internalization ability and cytotoxicity than the smaller ones (2.5Au@PT and 3.5Au@PT). Moreover, the experimental results against Trastuzumab target cells (SKBR-3) demonstrated that the cytotoxicity of 5Au@PT was closely related to pro-apoptotic protein increase, anti-apoptotic components decrease, survival-proliferation pathways downregulation and uptaking by cells via the activation of the classical receptor-mediated endocytosis. This work thus suggests an utmost importance of size control at the AuNPs when designing molecular-cancer delivery systems. Indeed, nonetheless the present method results suitable for AuNPs synthesis, the stability and conjugation with anticancer molecules depends on particles size, which affects also the its interaction with cancer cells. AuNPs unfortunately are not the optimal candidates for photothermal therapy (PTT) due to their limited absorption in the near infrared (NIR) region. In accordance with other studies, to overcome the size dilemma is possible to assemble small AuNPs into larger structures through controllable interparticle interaction by using biodegradable AGMA1-SH, with the final aim to enhance the absorption of NIR light. Overall, this project should offer important data for particle design improvement and for speeding up AuNPs application in breast cancer therapy with enhanced and personalized therapeutic outcomes.

## Data Availability Statement

The raw data supporting the conclusions of this article will be made available by the authors to any qualified researcher on reasonable request.

## Author Contributions

LV, ER, and CD conceived the study, were responsible for the correctness of analyses, and contributed to writing and editing of the manuscript. All authors contributed to critical experiments and insights, participated to this manuscript writing, and approved the final manuscript.

## Conflict of Interest

The authors declare that the research was conducted in the absence of any commercial or financial relationships that could be construed as a potential conflict of interest.
