# Supplementary material for: Extra-Small Gold Nanospheres Decorated With a Thiol Functionalized Biodegradable and Biocompatible Linear Polyamidoamine as Nanovectors of Anticancer Molecules
Source: Front Bioeng Biotechnol. 2020 Mar 4;8:132. doi: 10.3389/fbioe.2020.00132 (PMC7065572; doi:10.3389/fbioe.2020.00132)
Supplement: Supplementary file 1 [file Presentation_1.pdf]

Supplementary Material

Extra-small gold nanospheres decorated with a thiol functionalized biodegradable and biocompatible linear polyamidoamine as nanovectors of anticancer molecules

Nora Bloise,[1,2] Alessio Massironi,[3] Cristina Della Pina,[4]*Jenny Alongi,[5] Stella Siciliani,[6] Amedea Manfredi,[5] Marco Biggiogera,[6] Michele Rossi,[4] Paolo Ferruti,[5] Elisabetta Ranucci,[5*] Livia Visai,[1,2 *]

[1]Department of Molecular Medicine (DMM), Biochemistry Unit, Center for Health Technologies (CHT), UdR INSTM University of Pavia, Viale Taramelli 3/B, 27100, Pavia, Italy

[2]Department of Occupational Medicine, Toxicology and Environmental Risks, Istituti Clinici Scientifici Maugeri S.p.A, IRCCS, Via S. Boezio 28, 27100, Pavia, Italy

[3]Department of Chemistry and Industrial Chemistry, dBIOlab Research Group, University of Pisa, UdR INSTM Pisa, Via Moruzzi 13, 56124 Pisa, Italy

[4]Dipartimento di Chimica, Università degli Studi di Milano e CNR-ISTM, Via C. Golgi 19, 20133, Milano, Italy

[5]Dipartimento di Chimica, Università degli Studi di Milano, Via C. Golgi 19, 20133 Milano, Italy

[6]Department of Biology and Biotechnology, University of Pavia, Via Ferrata 9, 27100, Pavia, Italy

*** Correspondence:**
Corresponding Author
cristina.dellapina@unimi.it
elisabetta.ranucci@unimi.it
livia.visai@unipv.it

Supplementary Figures

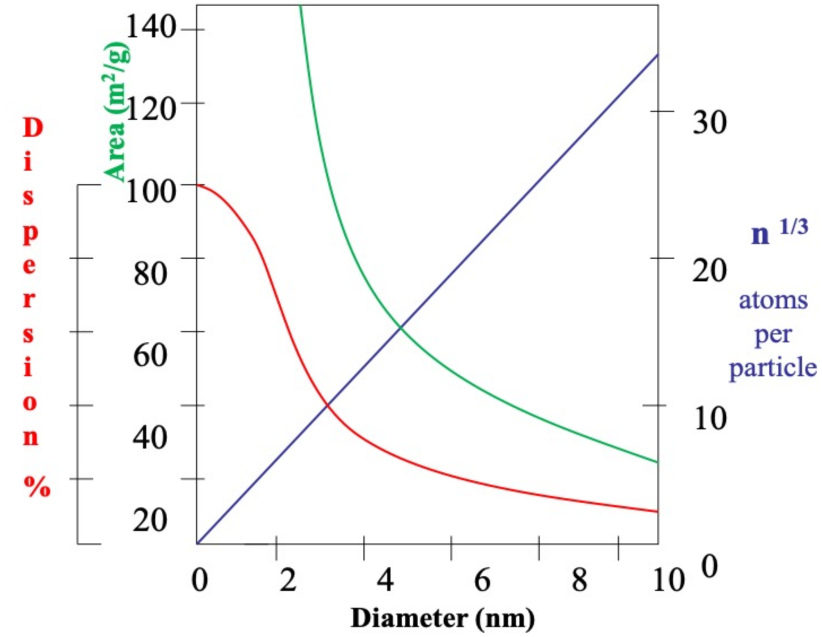

Figure S1. Determination of gold dispersion

Table S1. Synthesis of gold nanoparticles decorated with AGMA1SH and Trastuzumab

Sample	$HAuCl_4$ [10 mg/mL]		AGMA1-SH [10 mg/mL]		Trastuzumab [20 mg/mL]		$NaBH_4$ [10 mg/mL]		MilliQ water
	[mL]	[mg]	[mL]	[mg]	[mL]	[mg]	[mL]	[mg]	[mL]
2.5Au@PT	0.1	1.0	0.1	1.0	0.05	1.0	0.1	1.0	49.65
3.5Au@PT	0.5	5.0	0.5	5.0	0.25	5.0	0.5	5.0	23.25
5Au@PT	1.0	10.0	1.0	10.0	0.5	10.0	1.0	10.0	21.50
2.5Au@P	0.1	1.0	0.1	1.0			0.1	1.0	49.70
3.5Au@P	0.5	5.0	0.5	5.0			0.5	5.0	23.50
5Au@P	1.0	10.0	1.0	10.0			1.0	10.0	22.00
2.5Au@T	0.1	1.0			0.05	1.0	0.1	1.0	49.75
3.5Au@T	0.5	5.0			0.25	5.0	0.5	5.0	23.75
5Au@T	1.0	10.0			0.5	10.0	1.0	10.0	22.50

Table S2. Conjugation efficiency of AuNPs without AGMA1-SH by BCA analysis

Samples	conjugation efficiency %[a]	μg of Trastuzumab per 20 μg of nanoparticles[b]
2.5Au@T	25.0 ± 3.1	5.0 ± 0.6
3.5Au@T	31.1 ± 3.7	6.2 ± 0.2
5Au@T	38.9 ± 3.9	7.8 ± 0.8

[a] conjugation efficiency % = (1 – ([Trastuzumab in the supernatant]/[Trastuzumab added in the conjugation reaction])) × 100
[b] μg of Trastuzumab per 20 μg nanoparticles = Trastuzumab added in the conjugation reaction - Trastuzumab in the supernatant

Table S3. Dot blot assay conjugation efficiency quantification ofAuNPs with or without AGMA1-SH

Samples	μg of Trastuzumab per 20 μg of nanoparticles*
2.5Au@T	3.4 ± 0.5
3.5Au@T	4.3 ± 0.7
5Au@T	7.5 ± 0.3
2.5Au@PT	11.5 ±0.7
3.5Au@PT	15.2 ± 1.1
5Au@PT	17.5 ± 0.7

*calibration curve containing known amounts of Trastuzumab was used for determining the μg T per 20 μg of nanoparticles as reported in the *Experimental Section*

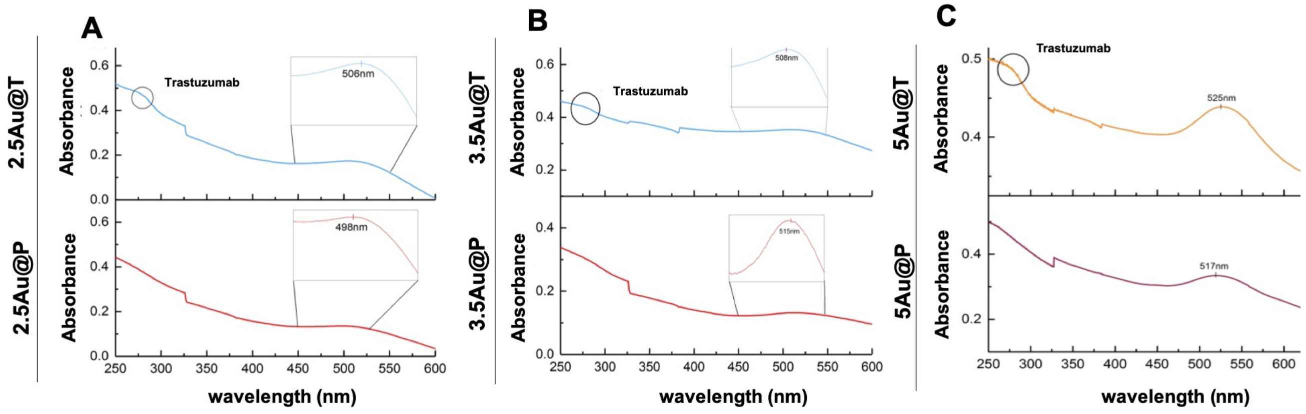

Figure S2. UV-vis absorbance spectra obtained from 2.5 Au@T, 3.5 Au@T and 5Au@T and from 2.5 Au@P, 3.5 Au@P and 5Au@P nanoparticles, respectively.

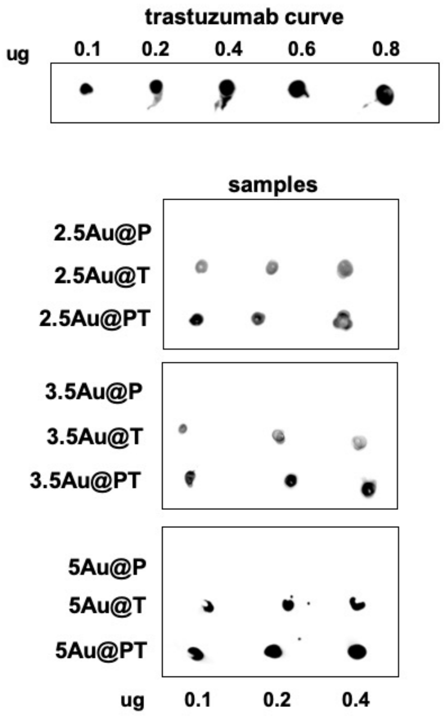

Figure S3. Dot blot assay of Trastuzumab amount conjugated to the different types of gold nanoparticles synthetized. Different amounts of Trastuzumab-functionalized Au@P differently sized were loaded on a nitrocellulose membrane. Trastuzumab (T) was used as positive control, whilst 2.5Au@P, 3.5A@P, 5Au@P were as negative controls (CTRL), respectively. The presence of T was detected by anti-human Horse Radish Peroxidase antibody only in 2.5Au@PT, 3.5Au@PT, and 5Au@PT spots and in positive control (T), confirming the linking of T to gold nanoparticles.

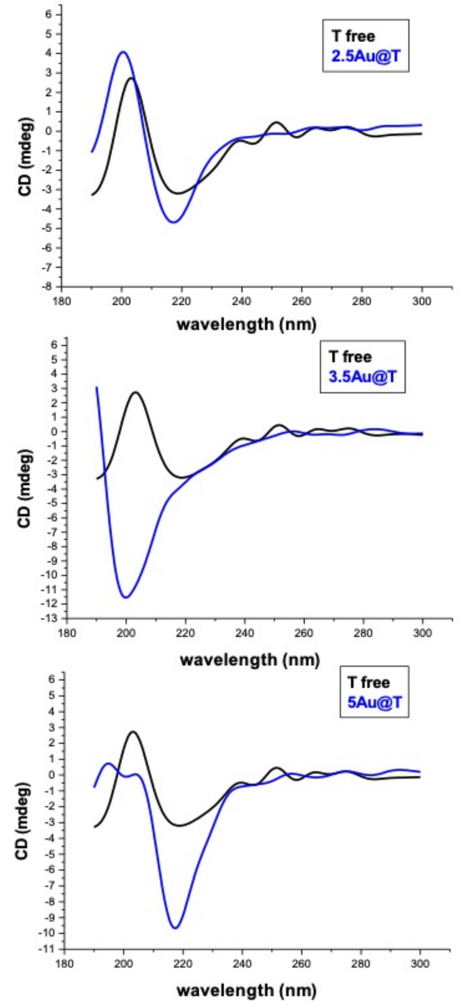

Figure S4. Circular dichroism spectra obtained from Trastuzumab free (black), and from 2.5 Au@T, 3.5 Au@T and 5Au@T nanoparticles (blue).

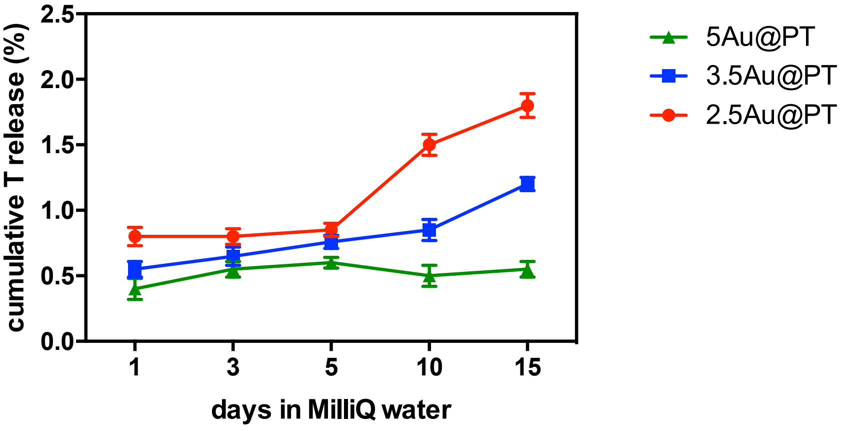

Figure S5. *In vitro* Trastuzumab release profile from 2.5Au@PT, 3.5Au@PT and 5Au@PT in MilliQ water at room temperature. Data are represented as cumulative percentage release at each time point relative to the conjugated-amount (at day 0) ± SD (n = 3).

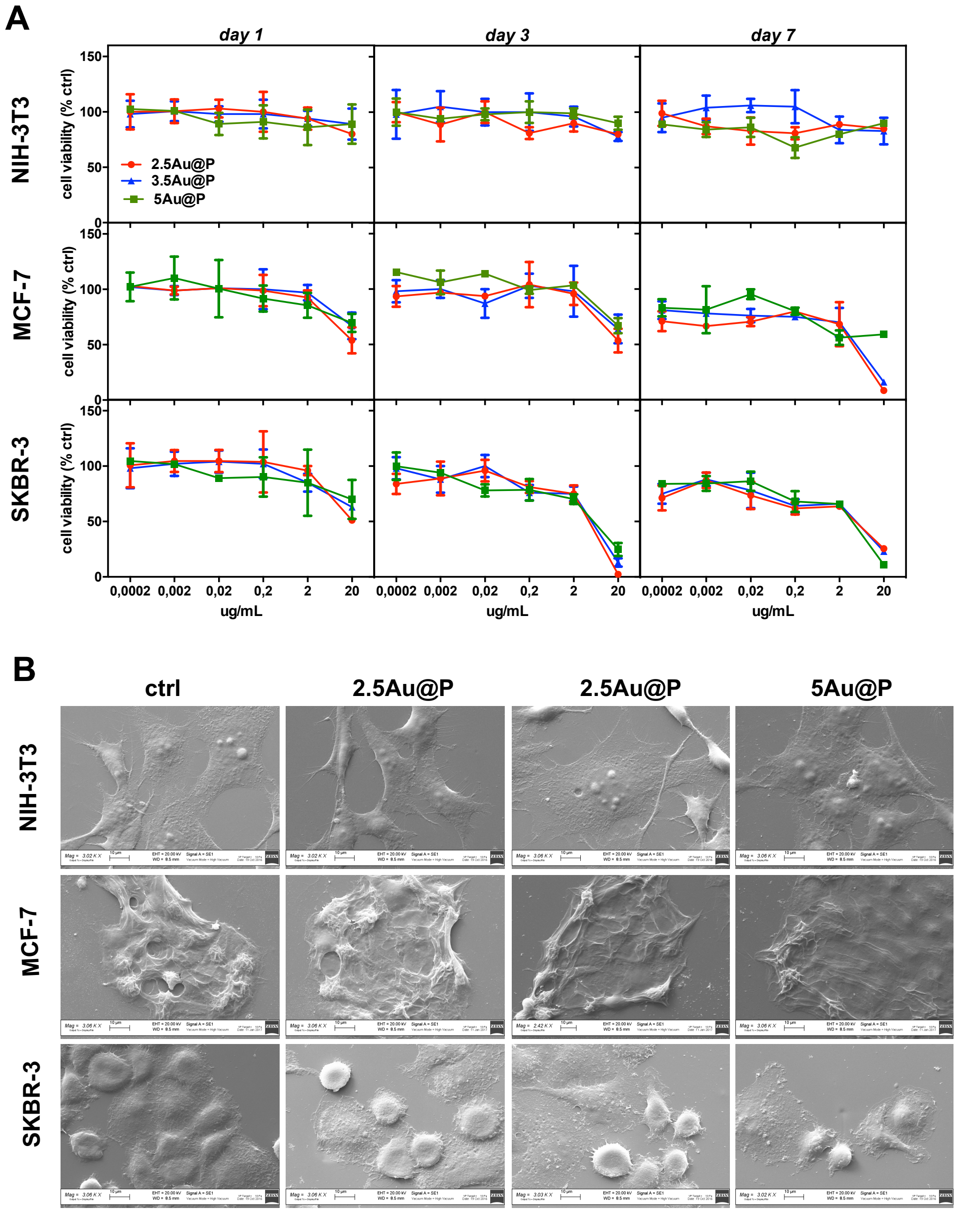

Figure S6. A) Cell viability assessment after incubation with unfunctionalized 2.5Au@P, 3.5Au@P and 5Au@P nanoparticles. **B)** SEM images obtained after 3 days of incubation with unfunctionalized 2.5Au@P, 3.5Au@P and 5Au@P nanoparticles. Mag = 3k×; scale bars = 10 µm.

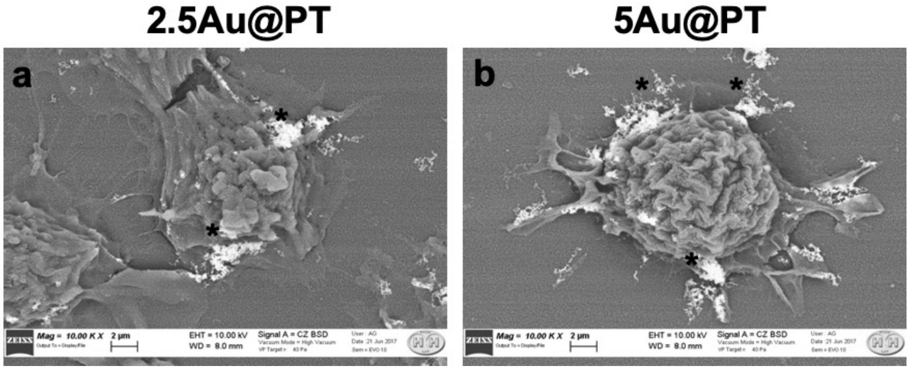

Figure S7. Representative images of 2.5Au@PT and 5Au@PT accumulation on SKBR-3 cells surface observed by SEM using the BS detector.

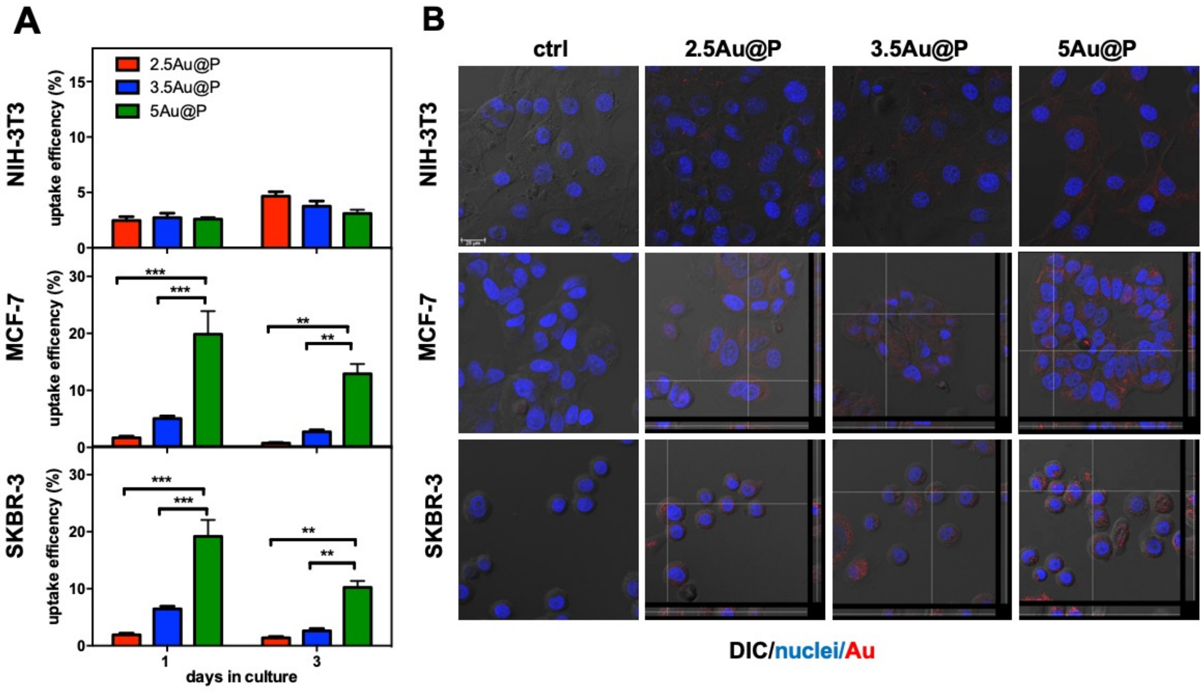

Figure S8. Internalization of 2.5Au@P, 3.5Au@P, and 5Au@P into cells. **A)** Uptake efficiency obtained by ICP-MS analysis at different time of incubation (** $p < 0.01$ and *** $p < 0.0001$). **B)** CLSM DIC (differential interference contrast) mode of cells exposed to the different nanoparticles Orthogonal view of images stacks is shown. Scattering of Au in red (false colour), nuclei in blue.

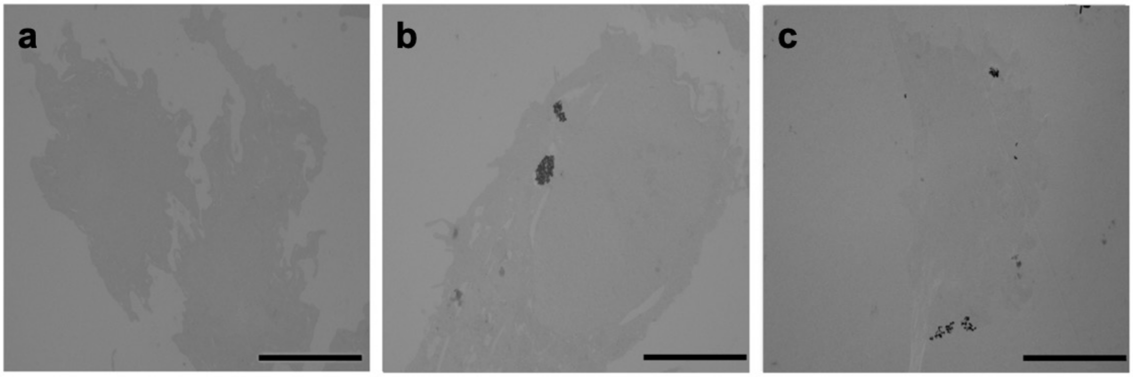

Figure S9. In order to rule out the possible interference of osmium, uranyl and lead precipitates, some samples were either fixed with aldehydes alone and the uranyl-lead staining omitted. a) Ctrl cell fixed with glutaraldehyde-osmium tetroxide; b) 5Au@PT treated cell fixed with glutaraldehyde-osmium tetroxide; c) 5Au@PT treated-cell fixed with glutaraldehyde alone; scale bars 5 μm.
